# Low-temperature thermodynamics with quantum coherence

**DOI:** 10.1038/ncomms8689

**Published:** 2015-07-03

**Authors:** Varun Narasimhachar, Gilad Gour

**Affiliations:** 1 Department of Mathematics and Statistics and Institute for Quantum Science and Technology, University of Calgary, 2500 University Drive NW, Calgary, Alberta, Canada T2N 1N4

## Abstract

Thermal operations are an operational model of non-equilibrium quantum thermodynamics. In the absence of coherence between energy levels, exact state transition conditions under thermal operations are known in terms of a mathematical relation called thermo-majorization. But incorporating coherence has turned out to be challenging, even under the relatively tractable model wherein all Gibbs state-preserving quantum channels are included. Here we find a mathematical generalization of thermal operations at low temperatures, ‘cooling maps', for which we derive the necessary and sufficient state transition condition. Cooling maps that saturate recently discovered bounds on coherence transfer are realizable as thermal operations, motivating us to conjecture that all cooling maps are thermal operations. Cooling maps, though a less-conservative generalization to thermal operations, are more tractable than Gibbs-preserving operations, suggesting that cooling map-like models at general temperatures could be of use in gaining insight about thermal operations.

## Abstract (web summary)

Thermal operations, a model of thermodynamic processes for small quantum systems out of equilibrium, are well-understood in absence of coherence. Here the authors introduce cooling processes, a generalization of thermal operations and find necessary and sufficient conditions for coherent state transitions via cooling processes.

## Graphical abstract

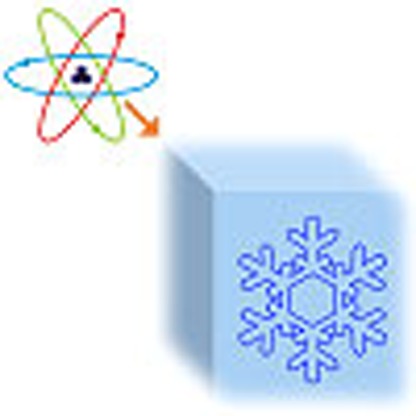

Advancements in cryogenics have enabled us to prepare systems at very low temperatures using various cooling techniques^1^,^2^,^3^. In fact, humans may soon cool systems to levels that are not known to exist anywhere in the observable universe! Low-temperature systems exhibit exotic, characteristically quantum phenomena such as the quantum Hall effect, superconductivity and topological order^4^,^5^,^6^, enabling diverse technological applications such as precision measurement instruments^7^,^8^, fast digital electronics^9^ and NMR applications^10^. One of the biggest potential applications is quantum computing—several of the proposed implementations of quantum computing are currently dependent on low-temperature capability^11^,^12^,^13^,^14^. In addition, low-temperature systems are useful in fundamental research frontiers such as particle physics^15^ and dark matter detection^16^.

The prevalence of such phenomena at low temperatures is related to the fact that coherence can better endure thermal noise at low temperatures^17^. On the other hand, significant strides have been made in realizing coherent quantum phenomena at higher temperatures^18^. These developments mean that more and more experimentally realizable systems exhibit effectively ‘low-temperature-like' behaviour at temperatures that are no longer forbiddingly low.

Our ability to control and manipulate physical systems in either of these cases—actual or effective low-temperature settings—hinges on our understanding of the thermodynamics of low-temperature environments. While classical thermodynamics is an adequate tool for analysing macroscopic systems in thermodynamic equilibrium, it proves inadequate in any situation involving microscopic quantum systems or thermodynamic non-equilibrium. There has been extensive interest in formulating a theory of thermodynamics applicable to such situations^19^,^20^,^21^,^22^,^23^,^24^,^25^,^26^,^27^,^28^,^29^,^30^,^31^,^32^,^33^,^34^. Most of these works, especially the recent ones, have studied thermodynamical processes using a model called ‘thermal operations', which are defined operationally as processes realizable by coupling a system with a heat reservoir and carrying out a global energy-conserving unitary evolution. However, existing formulations have not been able to fully incorporate quantum coherence—the essential aspect of quantum physics that is represented in the iconic ‘Schrödinger's cat' thought experiment. While coherence becomes irrelevant in the special case where the Hamiltonian of a system is fully degenerate^27^, it is essential to understand the thermodynamics of general systems. Moreover, coherence is a resource, helpful both in thermodynamic tasks such as work extraction^28^,^29^,^32^ and in other resource-based tasks such as reference frame alignment^35^. A recent surge of work in the field has made progress in understanding the role of coherence in thermodynamics^30^,^31^,^32^,^33^,^34^.

In ref. 33, the authors find an upper bound to the extent to which coherence can be preserved under thermal operations. In ref. 34, progress beyond such bounds has been made, but exact state transition conditions remain elusive. A possible strategy to gain further insight is to consider a set of processes beyond thermal operations, namely all quantum channels that preserve the Gibbs state. However, it is not clear if this expanded set is physically motivated, because it is defined mathematically rather than operationally.

In this paper, we report a mathematical generalization of coherent thermal operations at low temperatures, which we call the ‘cooling maps' model. At temperatures low enough for the ambient bath to be approximately in its ground state, thermal operations have the effect of taking away heat from the system of interest, therefore cooling the system. This motivates our definition of ‘cooling maps'. We find the necessary and sufficient condition for state transitions to be feasible under these maps. We construct thermal operation implementations for cooling maps that saturate the coherence transfer bounds of ref. 33, opening up the possibility of improvements in coherence-based tasks. Our work also sheds light on the relations between different models that could be used to study low-temperature quantum thermodynamics—thermal operations, Gibbs-preserving operations and our cooling maps (see Fig. 1). In general, cooling maps are potentially less conservative than thermal operations, in the sense that processes that are forbidden under thermal operations could be allowed under cooling maps. However, we demonstrate that the latter are much more conservative than Gibbs-preserving operations. Although the cooling maps model emerges from the low-temperature limit, the methods used in our work could potentially lead to a better understanding of coherence in quantum thermodynamics at all temperatures, in conjunction with the methods and results from other recent works that address this subject.

## Results

### Background: thermal operations

The physical setting in our model is a *d*-dimensional quantum system S whose free Hamiltonian is *H*_S_. For convenience, we make some simplifying assumptions on *H*_S_. First, that *H*_S_ has no degenerate energy levels. Thus, its energy spectrum has the structure


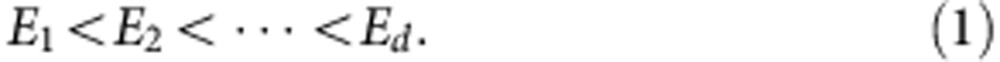


We also assume that *E*_*i*_−*E*_*j*_ ≠*E*_*k*_−*E*_*l*_ for any two pairs of indices (*i*,*j*) and (*k*,*l*), except when either *i*=*j* and *k*=*l*, or *i*=*k* and *j*=*l*.

Note that these assumptions are satisfied for almost all Hamiltonians, in a statistical sense: the subset of matrices that fail to satisfy these assumptions is of measure zero in the set of all Hermitian matrices. One may dismiss this measure-theoretic argument on the grounds that Hamiltonians of typical naturally occurring systems, such as atoms, have degenerate levels and gaps. But these degeneracies can be broken with the slightest perturbation, such as an external electromagnetic field. The absence of any such perturbation is in fact an exceptional circumstance, and it is reasonable to suppose that the above assumptions are satisfied by most realistic physical systems. Moreover, certain physical systems that are used in applications have these properties. For example, different types of superconducting artificial atoms used in quantum computing implementations, such as Cooper-pair boxes and transmons, are governed by anharmonic oscillator-like Hamiltonians^36^. Another important point to consider about these assumptions is the scope of their impact on our results. For systems that do satisfy these assumptions, the state transition conditions that we will derive turn out to be necessary and sufficient. However, even for systems that fail to satisfy these assumptions, our conditions remain sufficient, only losing their necessity. Furthermore, our results on maximally coherent processes hold regardless of these assumptions.

The non-degeneracy of all energy levels of *H*_S_ implies that we can label the eigenvectors (stationary states) using just one label, as in |*E*_*j*_〉. If S is isolated, its dynamics is governed by the Schrödinger equation under *H*_S_. If, instead, it is capable of exchanging heat with a thermal reservoir (heat bath) at temperature *T*, then S eventually ‘equilibrates', that is, approaches the state of thermal equilibrium with the reservoir, regardless of its initial state. The equilibrium state is given by the so-called Gibbs state


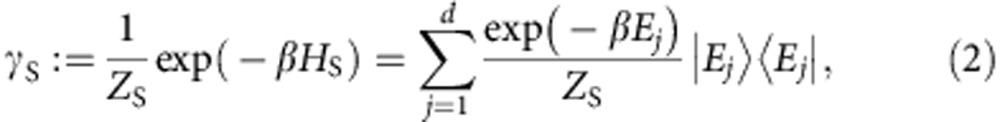


where *β*=(*k*_B_*T*)^−1^ with *k*_B_ the Boltzmann constant and 
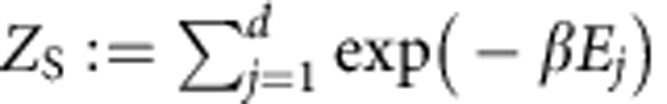
 the partition function of S.

Quantum thermodynamics enables us to go beyond just this asymptotic description and to determine what processes can occur in the course of equilibration. If the bath is ‘large' enough, every possible physical process occurring on the system S can be modelled through the following stepwise operational form:

1. Bring S (which is initially isolated) together with an arbitrary ancillary system A, which is prepared in its own Gibbs state *γ*_A_:=(1/*Z*_A_)exp(−*βH*_A_) corresponding to its own free Hamiltonian *H*_A_ and the ambient temperature *T*. Physically, the ancilla is all or part of the heat bath.
2. Perform any global energy-conserving unitary evolution *U* on the composite SA. Energy conservation is imposed through the commutator relation [*U*, *H*_SA_]:=0, where *H*_SA_ is the Hamiltonian that governs uncoupled evolution of the composite system SA:
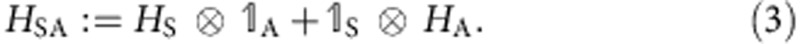
3. Discard the ancilla A (that is, isolate S again).

Mathematically, the process is represented by a completely positive trace-preserving map 
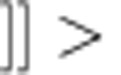

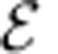
 whose action on an arbitrary state *ρ* of S is given by


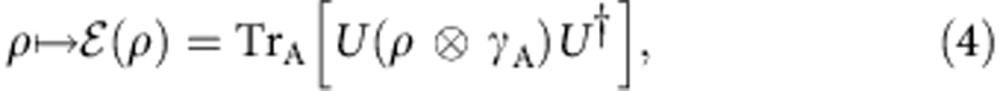


where 
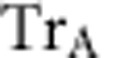
 is the mathematical operation of partial trace with respect to A, corresponding to the physical operation of discarding the system A.

Processes modelled in this manner have been called thermal operations in the literature (see Supplementary Note 1 for details). The energy conservation condition [*U*, *H*_SA_]:=0 can be understood in terms of the eigenvalues and eigenvectors of *H*_SA_: If {*G*_*j*_} are the eigenvalues of *H*_SA_, and |*G*_*j*_;*α*〉 represents an eigenvector belonging to *G*_*j*_ (where *α* could be a label identifying eigenstates within a degenerate energy level), then we require


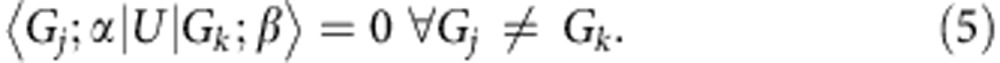


The uncoupled structure of *H*_SA_ means that its energy levels have the form *G*=*E*+*F*, where *E* and *F* are eigenvalues of *H*_S_ and *H*_A_, respectively. A unitary such as *U* can change the state of S by raising (lowering) *E* while simultaneously lowering (raising, respectively) *F* so as to keep *G* constant.

### The emergence of ‘cooling maps'

When the ambient bath temperature is low enough, the initial state of any ancilla A drawn from the bath (that is, its Gibbs state) is almost entirely in its lowest energy level:


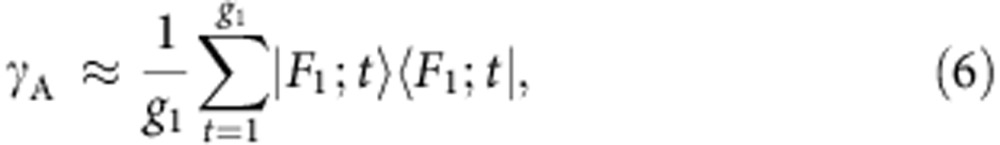


where *F*_1_ is the ground state energy, *g*_1_ the multiplicity of this energy level and *t* some label that identifies eigenvectors within the degenerate subspace. This means that even though the temperature is non-zero, the bath effectively behaves as though it were zero. Since the ancilla A starts out in its lowest energy level, any energy transfer that an energy-conserving unitary *U* causes between S and A must be from S to A. Therefore, the effect of a low-temperature thermal operation on S is to ‘cool' it. How low the temperature needs to be in order for this approximation to be valid is determined by the composition of the system and the bath. For example, a bath consisting of many identical systems in the same Gibbs state (that is, of the form *γ*^⊗*n*^) would satisfy this approximation at temperatures much lower than the gap between the ground and first excitated state of each subsystem. In some cases, one can infer this low-temperature behaviour of the bath indirectly, through the behaviour of the system of interest. For example, the ambient bath surrounding a superconducting artificial atom behaves effectively in this manner at temperatures lower than the superconducting critical temperature of the system. Under condition (6), together with our assumption of non-degenerate energy levels and gaps in *H*_S_, all thermal operations reduce to an elegant form, characterized by a Kraus operator sum representation with the following features: a number *n*≤*d* of diagonal Kraus operators


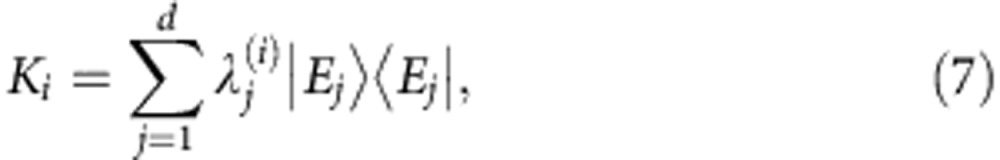


*i*∈{1…*n*}; and *d*(*d*−1)/2 Kraus operators of the form


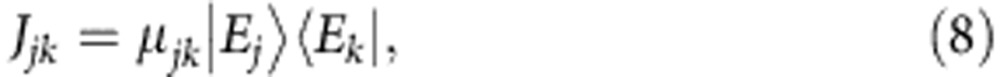


one for each pair (*j*,*k*) with *j*<*k*. Note that some of the *J*'s could be zero. If we relax the non-degeneracy conditions on the system Hamiltonian, the form of these Kraus operators generalizes to the well-known structure of amplitude-damping channels, which are used as a model of dissipation, spontaneous emission and so on^14^. The *j*<*k* condition in the *J*_*jk*_'s captures the ‘cooling' action that results from the low-temperature assumption. This motivates us to call any process with such an operator sum representation a ‘cooling maps'. A detailed derivation of this form may be found in Supplementary Note 2.

### The action of cooling maps

Let us denote by 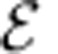 the channel realized by the above Kraus operators. The action of 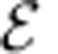 on the state of S can be expressed succinctly if we group the *λ*'s into *d* vectors of the form 
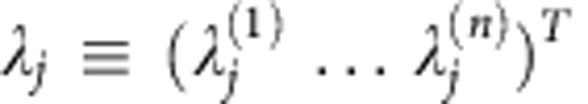
. If *ρ* is the initial state and *σ*=(*ρ*) the state after the application of 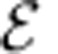, then the relation between the off-diagonal elements of *ρ* and *σ* is simple:


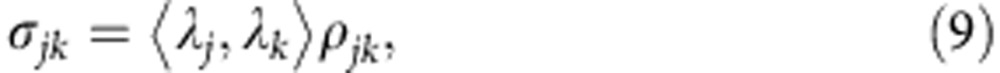


for each *j*≠*k*. Here 〈·,·〉 denotes the usual inner product between two vectors. On the other hand, the relation between the diagonal parts of the states is given by


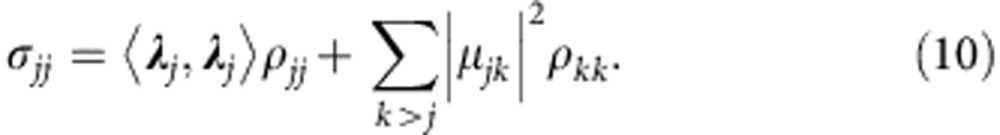


The matrix *q* whose components are the quantities *q*_*jk*_:=〈**λ**_*j*_,**λ**_*k*_〉 appearing above is called the Gramian of the collection {***λ***_*j*_}. Every Gramian matrix is positive-semidefinite, and conversely, every positive-semidefinite matrix is the Gramian of some collection of vectors^37^.

If we view the diagonal ***u***≡(*ρ*_11_…*ρ*_*dd*_)^*T*^ as a classical probability distribution, then its transformation under 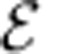 can be represented by the action of a matrix *P*:


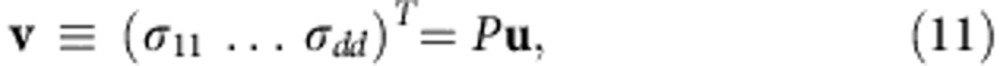


where the components of *P* are given by


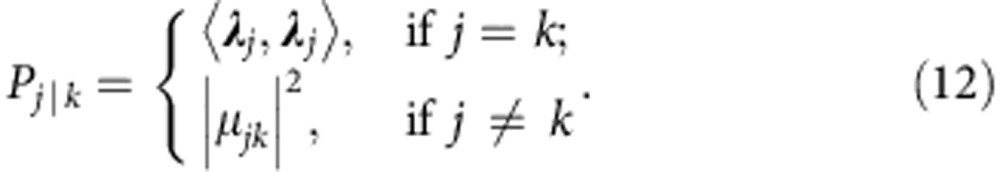


*P* is upper-triangular: *P*_*j*|*k*_=0 if *j*>*k*. Furthermore, it is column-stochastic: *P*_*j*|*k*_≥0 for all (*j*,*k*); and 
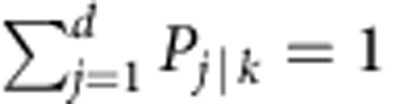
 for all *k*.

### Upper-triangular majorization

In Supplementary Note 2, we prove that the existence of an upper-triangular (UT) column-stochastic matrix *P* such that ***v***=*P**u*** is in fact equivalent to the simultaneous fulfillment of the following (*d*−1) inequalities:


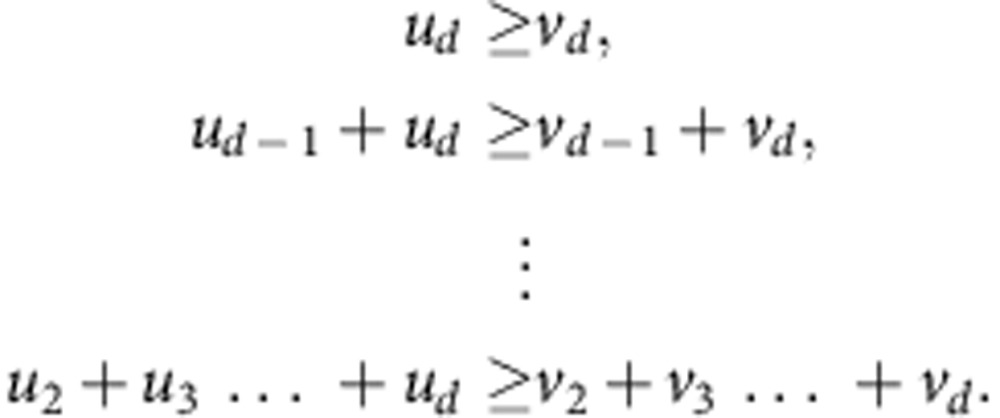


We abbreviate the above inequalities collectively as 
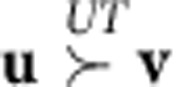
, read ‘***u*** UT-majorizes ***v***'. In the literature, UT majorization has variously been referred to as ‘unordered majorization'^38^ and ‘majorization'^39^ (not to be confused with the more common established sense of the term ‘majorization'), as well as the term we use^40^. It is instructive to compare UT majorization with the so-called thermo-majorization, which governs the transformation of the diagonal elements in thermodynamics at general temperatures^24^. The thermo-majorization relation between two probability distributions ***u*** and ***v*** can be defined in different ways, of which the following is perhaps most intuitive. Denote by ***u***_*γ*_ the Gibbs distribution for the given Hamiltonian at some inverse temperature *β*. That is,


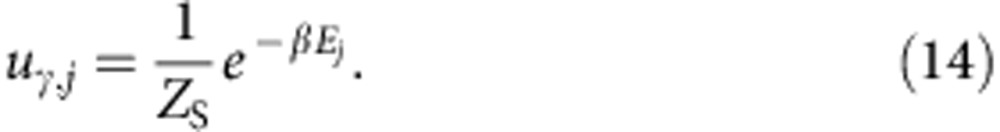


Then we say that ‘***u*** thermo-majorizes ***v***' if there exists a column-stochastic matrix *M* such that *M**u***_*γ*_=***u***_*γ*_, that is, *M* fixes the Gibbs distribution, and *M**u***=***v***. Considering that UT stochastic matrices fix the zero-temperature limit of the Gibbs distribution for a non-degenerate Hamiltonian, it seems intuitively reasonable that UT majorization emerges as the zero-temperature limit of thermo-majorization. We show this rigorously in Supplementary Note 2.

### State transformation conditions

The foregoing observations put together yield our main result—the necessary and sufficient condition for the feasibility of state transitions under cooling maps.

Theorem 1. Let *ρ* and *σ* be two states on S, arbitrary except that the matrix elements of *ρ* are non-zero (*ρ*_*jk*_≠0). Define the matrix *Q* as follows:


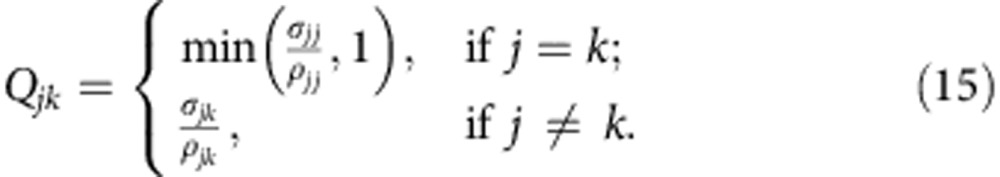


Then, the transition 
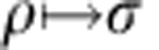
 is possible through a cooling map if and only if both the following conditions hold:

1. The diagonal parts ***u***≡(*ρ*_11_…*ρ*_*dd*_)^*T*^ and ***v***≡(*σ*_11_…*σ*_*dd*_)^*T*^ satisfy 
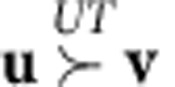
.
2. The matrix *Q* is positive-semidefinite: *Q*≥0.

The *Q* appearing above is in fact a special limiting case of the Gramian matrix *q* that we introduced earlier. Note that we can easily adapt the theorem to cases where some of the *ρ*_*jk*_'s are zero. Also note that if we relax the non-degeneracy assumptions on *H*_S_, the condition of this Theorem remains sufficient for state transitions; it is, however, no longer necessary. We provide the proof of this theorem, as well as technical details of the preceding discussion, in Supplementary Note 2.

### Optimally coherent cooling maps are thermal operations

We constructed the cooling maps based on the low-temperature limit of thermal operations. Since the latter link the mathematical model with actual physics, we must determine if the cooling and low-temperature thermal models are equivalent, or if instead there exist state transitions achievable by cooling maps but forbidden under thermal operations. A couple of special cases support the equivalence hypothesis.

The first special case is when S is a two-level system, that is, *d*=2, for which cooling maps are identical with thermal operations. This can be proved simply by constructing a thermal implementation of any cooling map (Supplementary Note 2). The state transition conditions for two-level systems under thermal operations at any temperature have been derived recently by Ćwikliński *et al.*^33^, and our result tallies with theirs in the low-temperature limit.

The other special case involves pairs of states (*ρ*, *σ*) satisfying the first condition of Theorem 1 and also


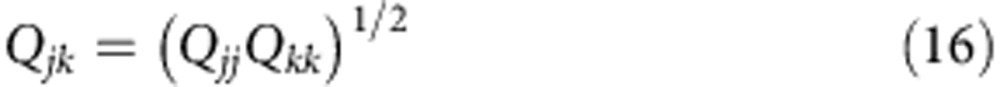


for all (*j*,*k*). Then there is a thermal operation taking 
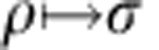
. The significance of this special case is that each off-diagonal element (that is, coherence between different energy levels) in *σ* has the highest magnitude possible, in the following sense. Suppose that 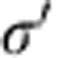 is a state whose diagonal coincides with that of *σ*. Then, if 
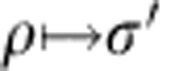
 is possible via a cooling map, then it holds for all (*j*,*k*) that 
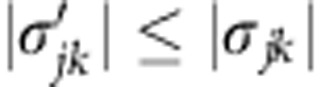
. We prove this bound in Supplementary Note 2. This bound was also proved for all temperatures in ref. 33, whose authors constructed examples where the bound cannot be attained. Our results show that it is always attainable at low temperatures. The same conclusion is reached in ref. 34, where the high-temperature case is also considered. More generally, we prove that any mixture of optimally coherent processes is a low-temperature thermal operation. In fact, this holds even if the non-degeneracy assumptions on the Hamiltonian *H*_S_ are relaxed.

### Gibbs-preserving operations

In general, the set of cooling maps could be larger than that of thermal operations. Whether the two sets are equivalent is an open problem. There is, however, an even larger set that includes both of these: all processes 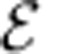 that preserve the Gibbs state *γ*_S_. That is, 
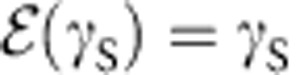
. These processes, called the ‘Gibbs-preserving operations', have been studied in the past as a possible model for thermodynamic processes. Even if one favours thermal operations as the physically more reasonable model, the Gibbs-preserving model can be studied as an approximation to thermal operations that is potentially more mathematically tractable. It is not hard to verify that all cooling maps are Gibbs preserving. We model the low-temperature limit for the Gibbs-preserving operations through the approximation 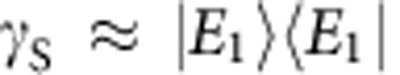. This form follows from our non-degeneracy assumption on the system Hamiltonian *H*_S_. Note that we do not require S to actually be in the Gibbs state; we merely require the ambient temperature to be low enough for the Gibbs state to be approximately equal to the ground state. Considering this approximate form of the Gibbs state, low-temperature Gibbs-preserving operations are processes 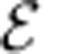 such that


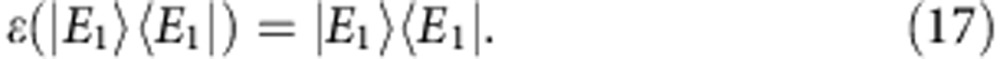


### Monotones under Gibbs-preserving operations

Clearly, the structure equation (17) of Gibbs-preserving operations privileges the *E*_1_ energy level in relation to the rest of the state space, leading to the following canonical parametrization of a generic state:


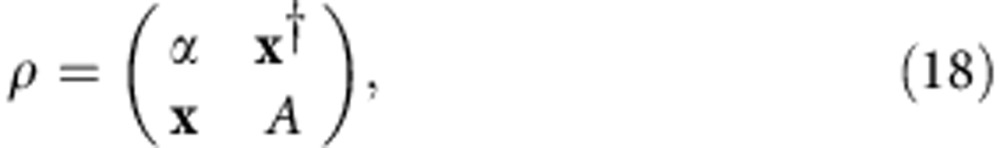


where *α*:=〈*E*_1_|*ρ*|*E*_1_〉≥0 is a real scalar, ***x*** is a complex (*d*−1)-dimensional vector, and *A* is a (*d*−1)-dimensional subnormalized density operator. In fact, any *ρ* can be reversibly converted (through an allowed unitary) to a state with a diagonal *A* and non-negative real entries in ***x***. The parameter *α* assumes its greatest value 1 when *ρ* coincides with the Gibbs state *E*_1_, and its least value 0 when *ρ* is supported on the subspace orthogonal to *E*_1_. Therefore, we can think of


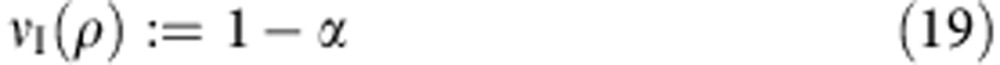


as a measure of the deviation of *ρ* from equilibrium, or in other words, its ‘non-equilibrium' (hence the letter *ν*). However, this measure does not contain any information about the coherences between different energy levels: it measures the non-equilibrium manifest in the diagonal part of *ρ*, related to the statistical distribution of energy amongst different energy levels. This aspect of non-equilibrium has in the past been referred to as ‘informational non-equilibrium'^27^ (hence the subscript ‘I').

Another measure of non-equilibrium is the quantity (We explain in Supplementary Note 3 how to assign a meaningful value to this quantity when A is singular.)


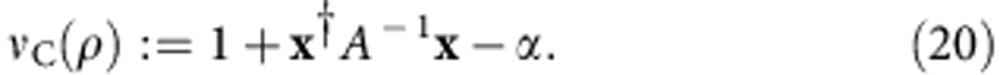


This quantity is also zero when *ρ*=*γ*_S_, and non-zero for other states. However, it relates with the coherences present in the state (hence the subscript ‘C'). The following result formalizes these quantities as measures of non-equilibrium.

Theorem 2. *ν*_I_ and *ν*_C_ are non-increasing under low-temperature Gibbs-preserving operations.

These quantities, which are among a more general family described in refs 31, 34, are examples of monotones under the allowed operations. They can be identified by characterizing the Kraus operator representations of Gibbs-preserving operations (details in Supplementary Note 3). In fact, since all cooling maps are Gibbs preserving, these quantities are monotones also under cooling maps and low-temperature thermal operations.

These monotones together constitute sufficient conditions for state transitions under low-temperature Gibbs-preserving operations in the case where S is a two-level system, that is, *d*=2. They also turn out to be sufficient when both *ρ* and *σ* are pure.

### Comparing different thermodynamical models

**Figure 2:**
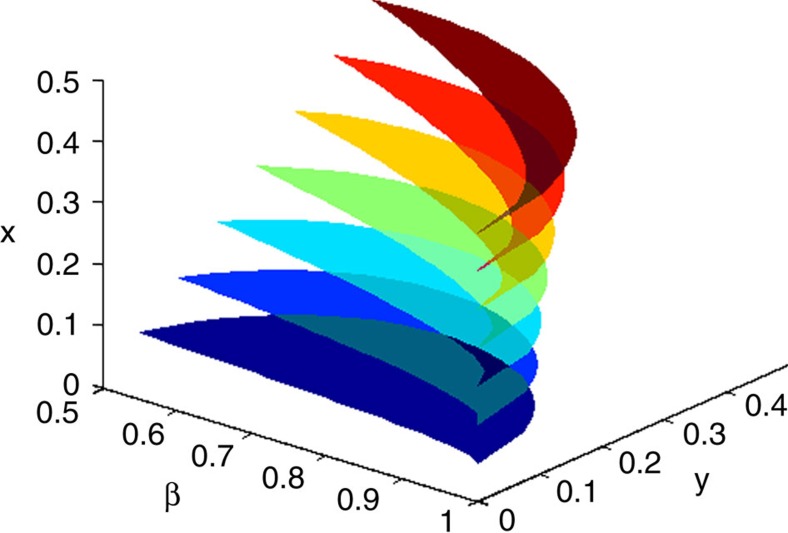
Gibbs-preserving operations 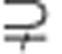 cooling maps. Consider a parametric family of initial states 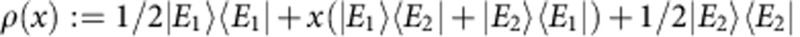, and a two-parameter family of final states 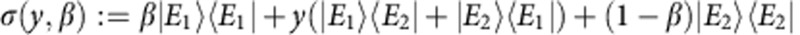, on a two-level system, with *x*,*y*,*β* real and non-negative. For each value of *x*, the corresponding region in the (*y*,*β*) plane represents part of the parametric state space that is reachable via Gibbs-preserving operations, but not via cooling maps (or thermal operations), from the initial state *ρ*(*x*).

In particular, the two-level case provides a platform (see Fig. 2) to compare the Gibbs-preserving model with the exact treatment of thermal operations (which are equivalent to cooling maps for two-level systems). A host of state transitions that are forbidden under thermal operations are nonetheless allowed under Gibbs-preserving operations. This implies that the monotones *ν*, when applied to thermal operations, are strictly less informative than the conditions of Theorem 1. The gaping disparity between the two models, which was first demonstrated in the recent work of Faist *et al.*^41^, brings to the fore an important question in this field: which of the two models is a more accurate description of reality? While that dilemma remains, we now know the exact state transition conditions for cooling maps, which are demonstrably closer to low-temperature thermal operations than Gibbs-preserving operations are. Therefore, if one were to consider thermal operations the best available thermodynamical model, and if one considered the Gibbs-preserving model as an approximation thereto, then our work shows that we could have a better shot at finding exact state transition conditions by exploring classes of processes (such as our cooling maps) that are better approximations than the Gibbs-preserving model.

## Discussion

Much remains to be discovered in the world of quantum thermodynamics. In particular, low-temperature situations, wherein exotic coherent phenomena lead to numerous technological applications, call for a thorough understanding of quantum coherences in thermodynamic processes. Some existing works on this aspect^28^,^32^ pertain to the use of environmental coherence to aid thermodynamic state transitions in the system, as opposed to the evolution of the system's own coherence under state transitions. Recent work on the latter^30^,^31^,^32^,^33^,^34^ provides insights that apply to all temperatures. Particularly, ref. 34 identifies the essence of this problem, namely the interplay between time-translation symmetry and thermal inequilibrium. However, in the general case, it appears to be challenging to find complete (necessary and sufficient) conditions that can be expressed succinctly. In this paper, we compromise on the range of temperatures in our scope of generality, but by doing so we make significant progress in the low-temperature regime through our ‘cooling maps' characterization. We find the necessary and sufficient conditions for state transitions under cooling maps, and also confirm rigorously that low-temperature thermal operations can optimally preserve coherences.

The main open question emerging from this work is whether the mathematically characterized cooling maps are equivalent to the physically motivated thermal operations, or merely a close approximation thereof. Their equivalence for the cases of two-level systems and mixtures of optimally coherent processes motivates us to conjecture equivalence in general. The study of cooling maps aided by catalysts, and possible generalizations to higher temperatures, are other open problems that would provide insight into thermodynamics. Likewise, the monotones derived from the Gibbs-preserving model could have higher-temperature generalizations that improve our understanding of coherence transfer in thermodynamics. Finally, there is potential for experimental realization and testing of our results, for instance using a superconducting artificial atom coupled with a network of spins that acts as a bath. We leave these avenues for future work.

## Additional information

**How to cite this article:** Narasimhachar, V. & Gour, G. Low-temperature thermodynamics with quantum coherence. *Nat. Commun.* 6:7689 doi: 10.1038/ncomms8689 (2015).

## Supplementary Material

Supplementary Information Supplementary Figures 1-2, Supplementary Notes 1-3 and Supplementary References

## Figures and Tables

**Figure 1:**
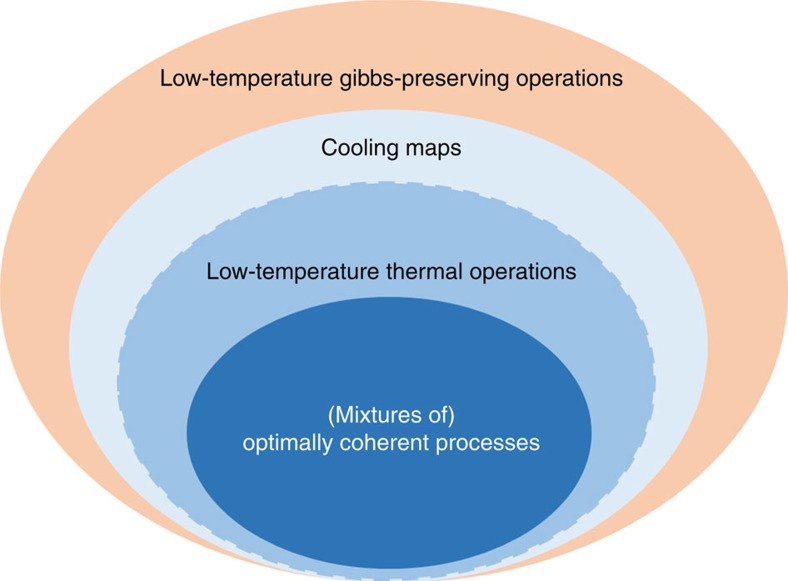
Inclusion hierarchy of thermodynamic models. In this work, we introduce the cooling maps as a generalization of low-temperature thermal operations, and the dashed boundary between the two sets indicates that the sets of state transitions they admit might coincide. Thermal operations include cooling maps that optimally preserve coherences.
