# Supplementary material for: Low-temperature thermodynamics with quantum coherence
Source: Nat Commun. 2015 Jul 3;6:7689. doi: 10.1038/ncomms8689 (PMC4506506; doi:10.1038/ncomms8689)
Supplement: Supplementary Information — Supplementary Figures 1-2, Supplementary Notes 1-3 and Supplementary References [file ncomms8689-s1.pdf]

Supplementary information

SUPPLEMENTARY FIGURE 1. PROOF IDEA FOR SUPPLEMENTARY LEMMA 2

A pictorial depiction of the construction of the stochastic map mapping $\boldsymbol{u} \mapsto \boldsymbol{v}$ in the proof of Supplementary Lemma 2. If $u_k \geq v_k$, then the kth (backwards!) step consists of mapping the fraction $P_{k|k} = v_k/u_k$ of u_k to complete the desired v_k and then adding the leftover part $(u_k - v_k)$ to a_{k+1} to enhance it to a_k.

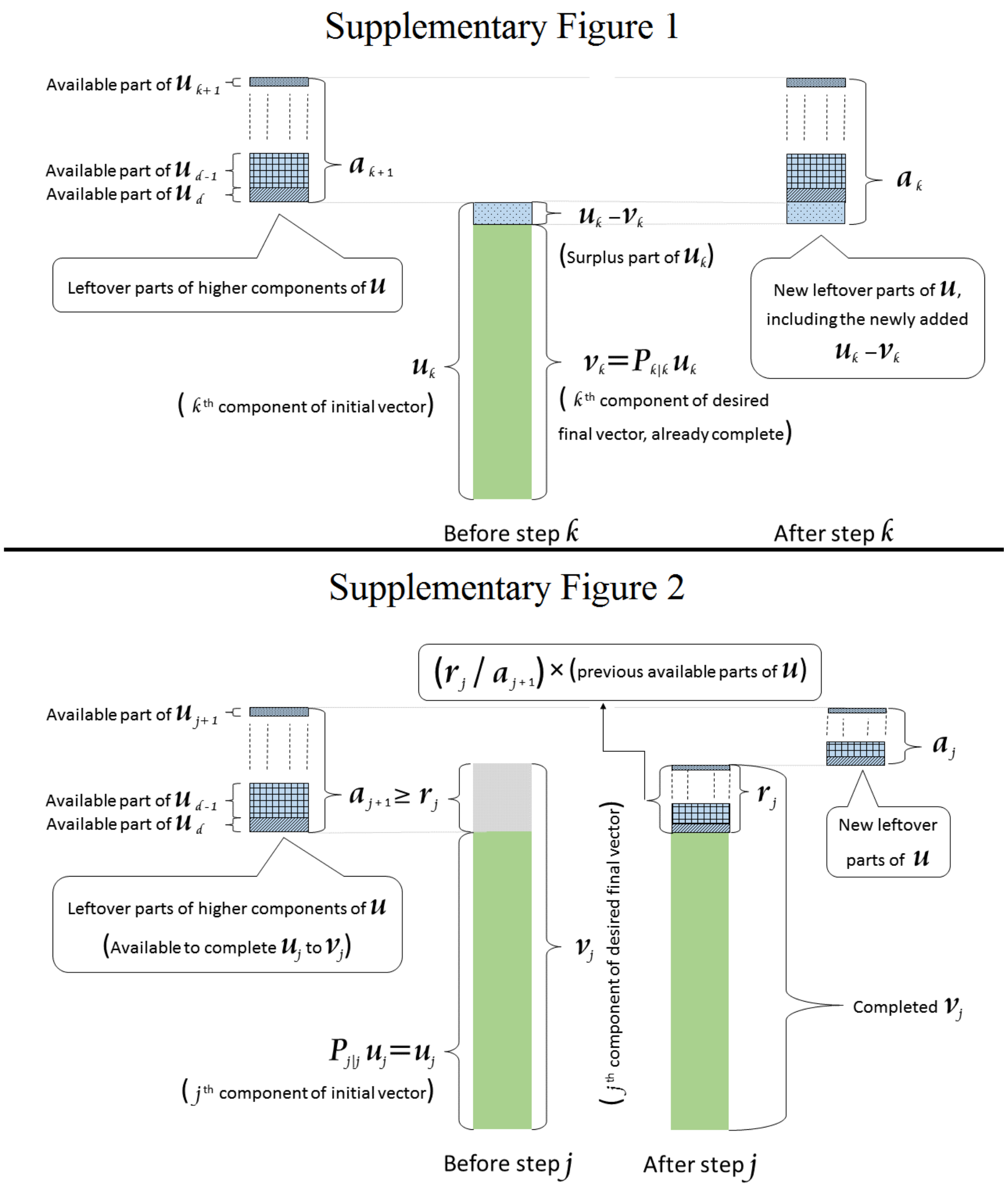

SUPPLEMENTARY FIGURE 2. PROOF IDEA CONTINUED

In the jth step, if $u_j < v_j$, then $P_{j|j} = 1$, and $P_{j|j} u_j = u_j$ is still smaller than the desired v_j by $r_j = v_j - u_j$. We then add (r_j/a_{j+1}) of the leftover part of each higher component of $\boldsymbol{u}$ to u_j to complete it to v_j.

SUPPLEMENTARY NOTE 1. THE THERMAL OPERATIONS MODEL

Here we provide a summary of the relevant background for understanding the "thermal operations" model of quantum thermodynamics. We base the discussion on the content of Refs. [1, 2].

Let us call the system of interest S. In classical thermodynamics, S is some composite system consisting of a huge number of constituent parts—a gas, a spin lattice, etc. In that case we can accurately model thermal properties using a formalism that does not actually monitor the exact quantum state (the "microstate") of S, but rather only a coarse-grained description that includes only a few so-called "macroscopic" variables, such as the temperature, pressure, and magnetic moment. On the other hand, in quantum thermodynamics, the microstate is part of the formalism. The "thermodynamic" element lies in how the *environment* is modeled: The environment is assumed to be an ideal thermal reservoir (or "heat bath"). This form of the environment, characterized by some properties that we will discuss below, naturally renders the dynamics of the system "thermalizing".

This approach allows us to not only match the classical thermodynamical expectation of eventual "equilibration" of the system with the environment, but to also understand how the microstate evolves *while the system equilibrates.* The processes that can occur in the course of equilibration are classified under the label "thermal operations".

The heat bath

The environment (call it R) of S is an ideal heat bath, characterized by the following properties:

1. The state of R is a Gibbs state at some temperature T. This temperature acts as the "ambient" condition determining the dynamics of S.
2. This state of R is supported almost entirely on a typical set $\mathcal{E}_{\mathrm{R}}$ of energy levels.
3. The energies F in the typical set $\mathcal{E}_{\mathrm{R}}$ are concentrated in a region of radius $O\left(F_M^{1/2}\right)$ around the mean value F_M. (We use F for energy levels of R, to distinguish them from those of S).
4. The multiplicity, or degeneracy, $g_{\mathrm{R}}(F)$ of energy levels in $\mathcal{E}_{\mathrm{R}}$ scales at least exponentially in F:
$$g_{\mathrm{R}}(F) \geq g_1 \exp\left[c_{\mathrm{R}}(F - F_1)\right] \tag{1}$$
for some constant $c_{\mathrm{R}} > 0$, where F_1 is the ground state energy.
5. For any two energies (E_i, E_j) of S, there exist $(F_k, F_\ell,)$ in $\mathcal{E}_{\mathrm{R}}$ such that
$$E_i - E_j = F_k - F_\ell. \tag{2}$$
6. For small perturbations about the peak F_M, the multiplicity goes as
$$g_{\mathrm{R}}(F_M - \epsilon) \approx g_{\mathrm{R}}(F_M) \exp(-\beta\epsilon), \tag{3}$$
where $\beta := 1/(k_B T)$ with k_B the Boltzmann constant.

All of these properties are exhibited by a system that consists of many weakly interacting identical systems all prepared in their respective Gibbs states, i.e., a composite in a state of the form $\gamma^{\otimes n}$ with γ a Gibbs state. In the present work, we are interested in the low-temperature limit. In this limit, the state of the bath is almost completely supported in its ground space, and therefore, all the above requirements excepting No. 5 are trivially satisfied.

Carrying out a thermal operation

We now consider the definition of thermal operations in detail, in order to clarify and justify the specifics. For convenience, we repeat below the definition of thermal operations from the text, with minor modifications.

Definition (Thermal operation). A process (i.e., a quantum channel) on S, that can be realized operationally in the following steps:

1. Bring S (which is initially isolated) together with an arbitrary ancillary system A, which is prepared in its own Gibbs state $\gamma_{\mathrm{A}} := (1/Z_{\mathrm{A}})\exp(-\beta H_{\mathrm{A}})$ corresponding to its own free Hamiltonian H_{A} and the ambient temperature T. Physically, A is all or part of the heat bath R, which in turn is modeled as discussed in the previous section.
2. Perform any global energy-preserving unitary evolution U on the composite system SA.
3. Discard the ancilla A (i.e., isolate S again).

Mathematically, the channel is represented by a completely positive (CP) trace-preserving (TP) map $\mathcal{E}$ whose action on an arbitrary state ρ of S is given by

$$\rho \mapsto \mathcal{E}(\rho) = \mathsf{Tr}_{\mathrm{A}}\left[U\left(\rho\otimes\gamma_{\mathrm{A}}\right)U^{\dagger}\right], \tag{4}$$

where Tr_{A} is the mathematical operation of partial trace with respect to A, corresponding to the physical operation of discarding the system A.

Let us look closely at the above operational description: What does it mean to be able to attach an *arbitrary* ancilla and perform an *arbitrary* energy-conserving unitary? The arbitrariness of the ancilla A means that the ancilla can feature *any* number of degrees of freedom, and that its free Hamiltonian H_{A} is unrestricted. H_{A} could even be time-dependent: as explained in Ref. [1], we can model time-dependence by a time-independent Hamiltonian, provided we include an additional "clock" system into the apparatus. But what about interactions between S and A? The fact that we start out and end up with S isolated implies that, while we can "turn on" an interaction in between, the initial and final settings must be ones where the dynamics of S is free. Therefore, the Hamiltonian of the composite SA at the start and end of the protocol has the form

$$H_{\mathrm{SA}} = H_{\mathrm{S}}\otimes\mathbb{1}_{\mathrm{A}} + \mathbb{1}_{\mathrm{S}}\otimes H_{\mathrm{A}}. \tag{5}$$

As explained in the main text, the energy conservation condition on the unitary evolution U can be stated in terms of the eigenvalues and eigenvectors of H_{SA} as

$$\langle G_j;\alpha|\,U\,|G_k;\beta\rangle = 0, \tag{6}$$

where G_j and G_k are distinct eigenvalues. Also recall from the main text that the energy levels of H_{SA} have the form

$$G_i = E_j + F_k, \tag{7}$$

where E_j is one of the eigenvalues of H_{S} and F_k an eigenvalue of H_{A}. An energy-conserving unitary can connect different energy levels on S by raising or lowering E while lowering or raising F by the same amount.

The low-temperature assumption

Here we make our notion of lowness of temperature more precise. We define low temperature with reference to the properties of the heat bath R, discussed earlier. One of the properties is that the state of the bath is a Gibbs state at some temperature T. This has the form

$$\begin{aligned}\gamma_{\mathrm{R}} &= \frac{1}{Z_{\mathrm{R}}}\exp\left(-\beta H_{\mathrm{R}}\right)\\ &= \sum_j \frac{\exp\left(-\beta F_j\right)}{Z_{\mathrm{R}}}\sum_{t=1}^{g_j}|F_j;t\rangle\langle F_j;t|\\ &= \sum_j \frac{g_j\exp\left(-\beta F_j\right)}{Z_{\mathrm{R}}}\Pi_j.\end{aligned} \tag{8}$$

Here we denote by g_j the multiplicity of level F_j, and t is some label that identifies individual eigenvectors within a degenerate subspace. $\Pi_j := (1/g_j) \sum_{t=1}^{g_j} |F_j;t\rangle \langle F_j;t|$ represents the normalized projector onto the subspace of energy F_j. If we now choose β large enough that

$$g_1 \exp(-\beta F_1) \gg g_j \exp(-\beta F_j) \tag{9}$$

for any $j \neq 1$, we then effectively have

$$\gamma_{\mathrm{R}} \approx \Pi_1, \tag{10}$$

which is the form in which the low-temperature assumption is used in the main matter. The range of temperatures at which this approximation is justified is determined by the nature of the bath, and also by the relation of the bath to the system. For example, for a bath consisting of many identical systems in identical Gibbs states, i.e. of the form $\gamma^{\otimes n}$, our low-temperature assumption is satisfied for temperatures $T \ll F_2 - F_1$, where F_1 and F_2 are the ground and first excited state energies of each subsystem in the bath. Interestingly, it might be possible to justify our low-temperature assumption even in cases where we know little about the actual composition of the bath: based on the behavior of the system itself. For example, if the system is a superconducting circuit and the bath is the environment that is not in our control, then at temperatures below the system's superconducting critical temperature one could assume the bath to be in its ground state. This is because the system's existence in the superconducting phase implies that no energy is flowing from the bath into the system. In the remainder, we will use the term "thermal operation" to mean "thermal operation under the low-temperature assumption".

SUPPLEMENTARY NOTE 2. CHARACTERIZING THERMAL OPERATIONS AS "COOLING MAPS"

As we discussed in the previous supplementary note, our low-temperature assumption leads to the property that the initial state of any ancillary system A used in implementing a thermal operation is supported almost entirely on its lowest energy level F_1:

$$\gamma_{\mathrm{A}} \approx \left(\frac{1}{g_1}\right) \sum_{t=1}^{g_1} |F_1;t\rangle \langle F_1;t| \,. \tag{11}$$

In this note we will see that this leads to a convenient mathematical model.

Cooling maps: motivation

Let us now turn our attention to the system of interest, S. It is characterized by its Hamiltonian H_{S}. Recall from the main text the following assumptions about H_{S}:

1. H_{S} has no degenerate energy levels. Thus, its energy spectrum has the structure

$$E_1 < E_2 < \cdots < E_d, \tag{12}$$

   where d is the number of degrees of freedom in S.

2. For any two pairs of indices, (i,j) and (k,l),

$$E_i - E_j \neq E_k - E_l, \tag{13}$$

   except when either $i=j$ and $k=l$, or $i=k$ and $j=l$.

These assumptions may seem very artificial and restrictive, but are in fact satisfied by generic physical systems. If a Hermitian matrix were chosen at random and assigned to act as the Hamiltonian, then with probability 1 it would have the above properties. One might argue that actual physical systems, such as atoms, don't occur with random Hamiltonians, and typically have degenerate levels and gaps. But these degeneracies exist only when the systems are perfectly isolated from all external influences (e.g. electromagnetic fields). In reality the degeneracies are broken, even if only by tiny perturbations. Furthermore, the gaps nature of such degeneracy-breaking phenomena, such as the Stark effect and the Zeeman effect,

Recalling Eq. (4), and using the approximation Eq. (11), we can write any thermal operation as

$$\begin{aligned}\mathcal{E}(\rho) &= \mathsf{Tr}_{\mathrm{A}}\left[U\left(\rho\otimes\gamma_{\mathrm{A}}\right)U^{\dagger}\right]\\ &\approx \mathsf{Tr}_{\mathrm{A}}\left[U\left(\rho\otimes\left[\frac{1}{g_1}\sum_{t=1}^{g_1}|F_1;t\rangle\langle F_1;t|\right]\right)U^{\dagger}\right]\\ &= \frac{1}{g_1}\sum_{t=1}^{g_1}\mathsf{Tr}_{\mathrm{A}}\left[U\left(\rho\otimes|F_1;t\rangle\langle F_1;t|\right)U^{\dagger}\right]\\ &= \frac{1}{g_1}\sum_{t=1}^{g_1}\mathcal{E}_t(\rho),\end{aligned} \tag{14}$$

where each $\mathcal{E}_t$ is a CPTP map defined through

$$\mathcal{E}_t(\rho) := \mathsf{Tr}_{\mathrm{A}}\left[U\left(\rho\otimes|F_1;t\rangle\langle F_1;t|\right)U^{\dagger}\right]. \tag{15}$$

The action of $\mathcal{E}_t$ is determined by the action of U on states of the form $|E_j\rangle\otimes|F_1;t\rangle$. In such a state, the energy of S is E_j while that of A is the lowest possible, F_1. An energy-conserving U can either retain the same amount of energy in either subsystem, or transfer some energy from S to A. Therefore, level j of S can be mapped only to levels $k\leq j$, and the overall effect is to "cool" S.

It is useful to characterize the $\mathcal{E}_t$'s through the structure of their Kraus operator decompositions. One possible set of Kraus operators $\{K_i\}$ can be constructed by assigning the following values to its matrix elements:

$$\langle E_j|\,K_i\,|E_k\rangle := \left(\left(\langle E_j|\otimes\langle v_i|\right)U\left(|E_k\rangle\otimes|F_1;t\rangle\right)\right), \tag{16}$$

where

$$\{|v_i\rangle\} = \{|F_\ell;s\rangle\}. \tag{17}$$

Physically, the above construction represents the fact that K_i can change the state of S from $|E_k\rangle\mapsto|E_j\rangle$ by virtue of U taking the composite SA from $|E_k\rangle\otimes|F_1;t\rangle\mapsto|E_j\rangle\otimes|v_i\rangle$. The K_i's thus constructed fall into two categories:

1. When $|v_i\rangle=|F_1;s\rangle$ for some s: This case corresponds to U not causing any flow of energy from S to A (since A stays within the same energy level where it started). Because H_{S} has no degeneracies, the final state of S, $|E_j\rangle$, must be identical with its initial state, $|E_k\rangle$. Therefore the K_i's in this category are *diagonal.*

2. When $|v_i\rangle=|F_\ell;s\rangle$ is an excited state of A: Here U is raising A from F_1 to $F_\ell\neq F_1$. Therefore, for energy conservation,

$$E_k-E_j=F_\ell-F_1. \tag{18}$$

By the property 2 of H_{S}, there must be a unique pair (j,k) satisfying this condition for a given ℓ. Therefore, only one matrix element of such a K_i can be non-zero, and so we arrive at the form

$$K_i\propto|E_j\rangle\langle E_k|. \tag{19}$$

In the second category, note that j is always smaller than k. Since each of the $\mathcal{E}_t$'s can be Kraus-decomposed in this way, and $\mathcal{E}$ is an incoherent mixture of the $\mathcal{E}_t$'s, such a Kraus decomposition also exists for $\mathcal{E}$. This suggests that probing the set of all channels with such Kraus decompositions might shed light on thermal operations. To this end, we define

Definition (Cooling map). A quantum channel (CPTP map) with a Kraus decomposition consisting of Kraus operators of the following two classes:

1. Diagonal matrices $\{K_1\ldots K_n\}$. Without loss of generality, we can assume $n\leq d$.

2. Matrices of the form $J_{jk}\propto|E_j\rangle\langle E_k|$, $j<k$. Without loss of generality we can assume that there is only one J for every index pair (j,k). For, if $\mu_{jk}|E_j\rangle\langle E_k|$ and $\nu_{jk}|E_j\rangle\langle E_k|$ are two Kraus operators occurring in the same decomposition of some channel, then we can combine them into just the one operator $\sqrt{|\mu_{jk}|^2+|\mu_{jk}|^2}\,|E_j\rangle\langle E_k|$.

All matrix representations are in the standard basis $\{|E_j\rangle\}$. Note that the elements of the matrices can be complex. By the discussion preceding the above definition, we have the following:

Observation 1. All low-temperature thermal operations are cooling maps.

The action of cooling maps

Let us examine the action of a generic cooling map $\mathcal{E}$ on a generic initial state ρ. Let a possible set of Kraus operators for $\mathcal{E}$ be

$$K_i = \begin{pmatrix} \lambda_1^{(i)} & 0 & \dots & 0 \\ 0 & \lambda_2^{(i)} & 0 & \vdots \\ \vdots & 0 & \ddots & 0 \\ 0 & \dots & 0 & \lambda_d^{(i)} \end{pmatrix}, \; i \in \{1 \dots n\};$$

$$J_{jk} = \mu_{jk} \left|j\right\rangle \left\langle k\right|, \; j < k \in \{1 \dots d\}. \tag{20}$$

Denote by $\boldsymbol{\lambda}_j$ the n-dimensional complex vector whose components are $\lambda_j^{(i)}$. Let q be the *Gramian matrix* of the collection $(\boldsymbol{\lambda}_1 \dots \boldsymbol{\lambda}_d)$ of vectors. The Gramian is defined through

$$q_{jk} = \left\langle \boldsymbol{\lambda}_j, \boldsymbol{\lambda}_k \right\rangle, \tag{21}$$

where on the right-hand side is the usual inner product between two vectors on $\mathbb{C}^n$. Define also the matrix $P \equiv (P_{j|k})$, through

$$P_{j|k} = \begin{cases} q_{jj}, & \text{if } j = k; \\ \left|\mu_{jk}\right|^2, & \text{if } j \neq k. \end{cases} \tag{22}$$

It can be seen by inspection that the action of $\mathcal{E}$ on ρ yields the state σ whose components are given by

$$\sigma_{jk} = \begin{cases} \sum_{\ell=1}^{d} P_{j|\ell}\rho_{\ell\ell}, & \text{if } j = k; \\ q_{jk}\rho_{jk}, & \text{if } j \neq k. \end{cases} \tag{23}$$

The matrix P has the following properties:

1. Upper-triangularity: $P_{j|k} = 0$ if $j > k$. This follows from the upper-triangularity of the J's.

2. Column-stochasticity: $P_{j|k} \geq 0$ for all (j,k); and $\sum_{j=1}^{d} P_{j|k} = 1$ for all k. The latter follows from the trace-preserving (TP) condition on the action of $\mathcal{E}$ [Eq. (23)]. The stochasticity of P is the motivation for our use of "conditional probability" notation to denote its matrix elements.

In connection with the Gramian of a set of vectors, we recall the following useful result from linear algebra [3]: For any collection $(\boldsymbol{v}_j)$ of vectors on an inner product space, the Gramian matrix q of the collection is positive-semidefinite. Conversely, any positive-semidefinite matrix is the Gramian of some collection of vectors. Combining this fact with the preceding observations about the action of cooling maps leads to:

Supplementary Lemma 1. For any two states (ρ, σ) of S, the existence of a cooling map $\mathcal{E}$ mapping $\rho \mapsto \sigma$ is equivalent to the existence of a $d \times d$ positive-semidefinite matrix q with the following properties:

1. The diagonal of q must be identical with the diagonal of an upper-triangular column-stochastic matrix P such that

$$(\sigma_{11} \dots \sigma_{dd})^T = P(\rho_{11} \dots \rho_{dd})^T. \tag{24}$$

2. Each off-diagonal element q_{jk} must satisfy

$$\sigma_{jk} = q_{jk}\rho_{jk}. \tag{25}$$

Upper-triangular stochastic matrices and majorization

It will be useful for our present purpose to better understand upper-triangular column-stochastic (UTCS) matrices. General column-stochastic matrices are known to induce a *preorder* on the set of probability distributions, called the *majorization* preorder [4]. In the following lemma, we prove that the action of UTCS matrices induces a *partial order*, which by analogy we name "upper-triangular majorization", or "UT majorization".

Definition (UT majorization). Let $\boldsymbol{u} \equiv (u_1, u_2 \dots u_d)^T$ and $\boldsymbol{v} \equiv (v_1, v_2 \dots v_d)^T$ be two d-dimensional probability distributions. We say that $\boldsymbol{u}$ *UT-majorizes* $\boldsymbol{v}$, denoted $\boldsymbol{u} \overset{\text{UT}}{\succ} \boldsymbol{v}$, if the following $(d-1)$ inequalities are satisfied:

$$\begin{aligned} u_d &\geq v_d, \\ u_{d-1} + u_d &\geq v_{d-1} + v_d, \\ &\vdots \\ u_2 + u_3 \dots + u_d &\geq v_2 + v_3 \dots + v_d. \end{aligned} \tag{26}$$

Supplementary Lemma 2. If $\boldsymbol{u}$ and $\boldsymbol{v}$ are d-dimensional probability vectors and there exists a UTCS matrix P such that $\boldsymbol{v} = P\boldsymbol{u}$, then $\boldsymbol{u} \overset{\text{UT}}{\succ} \boldsymbol{v}$.

Conversely, if $\boldsymbol{u} \overset{\text{UT}}{\succ} \boldsymbol{v}$, then there exists a UTCS P such that $\boldsymbol{v} = P\boldsymbol{u}$. In fact, there exists such a P with the following specific values on its diagonal:

$$P_{j|j} = \begin{cases} \min\left(\frac{v_j}{u_j}, 1\right), & \text{if } u_j > 0; \\ 0, & \text{if } u_j = 0. \end{cases} \tag{27}$$

Proof. Assume first that there exists a UTCS P such that $\boldsymbol{v} = P\boldsymbol{u}$. Componentwise, we have

$$\begin{aligned} v_d =& P_{d|d} u_d; \\ v_{d-1} =& P_{d-1|d} u_d + P_{d-1|d-1} u_{d-1}; \\ &\vdots \\ v_1 =& P_{1|d} u_d + P_{1|d-1} u_{d-1} \dots + P_{1|1} u_1. \end{aligned} \tag{28}$$

The stochasticity of P implies that each of its elements is no greater than 1 (i.e., $P_{j|k} \leq 1$). Therefore, the first of the above equations implies that $v_d \leq u_d$. Adding the first two equations, we get $v_{d-1} + v_d \leq u_{d-1} + u_d$. Continuing in this manner, we have all the desired inequalities to prove $\boldsymbol{u} \overset{\text{UT}}{\succ} \boldsymbol{v}$.

Now to prove the converse, assume that $\boldsymbol{u} \overset{\text{UT}}{\succ} \boldsymbol{v}$. We shall construct a P with the desired properties. Firstly, we fix the diagonal elements of P as claimed in the Lemma statement:

$$P_{j|j} = \begin{cases} \min\left(\frac{v_j}{u_j}, 1\right), & \text{if } u_j > 0; \\ 0, & \text{if } u_j = 0. \end{cases} \tag{29}$$

By construction, these values lie in the interval $[0, 1]$ and so we're on track to construct a stochastic P. For each j, we require P to act in such a way that

$$v_j = P_{j|d} u_d + P_{j|d-1} u_{d-1} \dots + P_{j|j} u_j. \tag{30}$$

The last term of the RHS, $P_{j|j} u_j$, is already fixed by our definition of the diagonal element $P_{j|j}$. It remains to choose the $P_{j|k}$ for all $k > j$ in such a way as to satisfy the above equation. The freedom we have in this choice is characterized by the quantity

$$r_j = v_j - P_{j|j} u_j = \max\left(0, v_j - u_j\right), \tag{31}$$

which we may think of as a "remainder" or "deficit": the part of the RHS of Eq. (30) that remains to be filled in. Now let us consider each j in sequence, starting from $j = d$.

The premise $\boldsymbol{u} \overset{\text{UT}}{\succ} \boldsymbol{v}$ implies that

$$u_d \geq v_d, \tag{32}$$

and therefore,

$$r_d = 0. \tag{33}$$

This means that Eq. (30) has been achieved for $j = d$. The part of u_d that is still "available" to be mapped to lower components of $\boldsymbol{v}$ is

$$a_d := u_d \left(1 - P_{d|d}\right) = u_d - v_d \geq 0. \tag{34}$$

Now consider $j = d-1$. Again, $\boldsymbol{u} \overset{\mathrm{UT}}{\succ} \boldsymbol{v}$ implies

$$\begin{aligned} u_{d-1} + u_d &\geq v_{d-1} + v_d. \\ \Rightarrow v_{d-1} - u_{d-1} &\leq a_d. \end{aligned} \tag{35}$$

But the deficit in the $(d-1)^{\text{th}}$ component is

$$r_{d-1} = \max\left(0, v_{d-1} - u_{d-1}\right) \leq a_d. \tag{36}$$

Therefore, this deficit can be filled in by some part of a_d. We do this by assigning

$$P_{d-1|d} := \frac{r_{d-1}}{a_d}\left(1 - P_{d|d}\right). \tag{37}$$

The components of P assigned thus far have taken care of Eq. (30) for $j = d$ and $j = d-1$. The part of $(u_{d-1} + u_d)$ that is still available to be mapped to lower components of $\boldsymbol{v}$ is

$$\begin{aligned} a_{d-1} &:= u_d\left(1 - P_{d|d} - P_{d-1|d}\right) + u_{d-1}\left(1 - P_{d-1|d-1}\right) \\ &= u_d + u_{d-1} - v_d - v_{d-1}, \end{aligned} \tag{38}$$

and again, $\boldsymbol{u} \overset{\mathrm{UT}}{\succ} \boldsymbol{v}$ implies that $a_{d-1} \geq 0$.

In the next step we have again that

$$r_{d-2} \leq a_{d-1} \tag{39}$$

and can therefore carry out a similar procedure as before, assigning

$$P_{d-2|d} := \frac{r_{d-2}}{a_{d-1}}\left(1 - P_{d|d} - P_{d-1|d}\right) \tag{40}$$

and

$$P_{d-2|d-1} := \frac{r_{d-2}}{a_{d-1}}\left(1 - P_{d-1|d-1}\right). \tag{41}$$

The basic idea is the following: for any k, if $u_k \geq v_k$, then $r_k = 0$ and $P_{k|k} u_k = v_k$, therefore we do not need to map any higher component (u_ℓ for $\ell > k$) to "complete" v_k. We can in fact add the surplus part $u_k - v_k$ to the "available" a_{k+1} to get a larger number, a_k, that is now available to complete the v_j's for $j < k$. Supplementary Figure 1 illustrates this idea.

On the other hand, if for some j, $u_j < v_j$ (Supplementary Figure 2), then $r_j > 0$ and $P_{j|j} u_j < v_j$. But in such a case, thanks to the UT majorization condition, we are assured that the "available" part left over from higher components, which by our convention we call a_{j+1}, is at least r_j. We then take the overall part left over from each higher component of $\boldsymbol{u}$ and use up exactly the fraction r_j / a_{j+1} of it to complete the jth instance of Eq. (30), i.e.,

$$v_j = \sum_{k \geq j} P_{j|k} u_k. \tag{42}$$

P is upper-triangular by construction. Furthermore, using the recursive definition of the components of P, we can verify that

$$P_{j|k} \geq 0 \tag{43}$$

and

$$\sum_{k \leq j} P_{k|j} = 1, \tag{44}$$

guaranteeing stochasticity.

Reality check: UT majorization emerges from thermo-majorization

In quantum thermodynamics at general temperatures, an ordering relation called *thermo-majorization* [2] plays the role corresponding to that of UT majorization in our formalism. Although we arrived at UT majorization through rigorously examining the energy conservation condition in low-temperature thermal operations, it is worth while to convince ourselves of the soundness of our low-temperature limit. Why this matter is not trivial will become clear when we consider the following definition of thermo-majorization:

Definition (Thermo-majorization). For d-dimensional probability distributions $\boldsymbol{u}$ and $\boldsymbol{v}$, $\boldsymbol{u}$ *thermo-majorizes* $\boldsymbol{v}$, denoted

$$\boldsymbol{u} \overset{\text{Th}}{\succ} \boldsymbol{v}, \tag{45}$$

if there exists a *column-stochastic* matrix P such that

1. P fixes the Gibbs distribution: $P\boldsymbol{u}_\gamma = \boldsymbol{u}_\gamma$, where $\boldsymbol{u}_\gamma := (1/Z_{\text{S}}) \left(\exp(-\beta E_1) \dots \exp(-\beta E_d)\right)^T$ is the diagonal part of the Gibbs state γ_{S}.
2. P maps $\boldsymbol{u}$ to $\boldsymbol{v}$: $\boldsymbol{v} = P\boldsymbol{u}$.

Ostensibly, it might seem that the low-temperature limit of thermo-majorization could be obtained by simply approximating the Gibbs state by the ground state:

$$\gamma_{\text{S}} \approx |E_1\rangle \langle E_1| \,. \tag{46}$$

This approximation would lead to a corresponding counterpart of thermo-majorization that is associated with all stochastic matrices P that obey

$$P_{j|1} = 0 \tag{47}$$

for $j > 1$. However, this is clearly different from UT majorization, which is associated with a more restricted class of such P's—namely, upper-triangular matrices. The following exercise serves to vindicate UT majorization as the right option in favour of the less-restrictive version. Consider some finite inverse temperature β. We then have the following conditions for P to fix $\boldsymbol{u}_\gamma$:

$$\begin{aligned}
P_{1|1} \exp(-\beta E_1) + \sum_{j>1} P_{1|j} \exp(-\beta E_j) &= \exp(-\beta E_1), \\
P_{2|1} \exp(-\beta E_1) + P_{2|2} \exp(-\beta E_2) + \sum_{j>2} P_{2|j} \exp(-\beta E_j) &= \exp(-\beta E_2), \\
&\vdots \\
\sum_{j<d} P_{d|j} \exp(-\beta E_j) + P_{d|d} \exp(-\beta E_d) &= \exp(-\beta E_d).
\end{aligned} \tag{48}$$

In the limit $\beta \to \infty$,

$$\exp\left[-\beta(E_j - E_k)\right] = 0 \tag{49}$$

whenever $j > k$. In this limit if we multiply the jth of Eqs.(48), for any $j > 1$, by $\exp(\beta E_1)$, we end up with

$$P_{j|1} = 0 \;\forall j > 1. \tag{50}$$

Now considering only the equations for $j > 2$, we multiply by $\exp(\beta E_2)$ to infer that

$$P_{j|2} = 0 \;\forall j > 2. \tag{51}$$

Proceeding in this manner, we can prove that P is upper-triangular in the limit.

One can carry out a similar verification with the other, equivalent definition of thermo-majorization in Ref. [2] (in terms of Gibbs-rescaled and reordered distributions). There one will find that for all distributions with no zero entries (i.e., for all but a measure-zero subset) the canonical permutation of vector components through which thermo-majorization is defined will approach the identity permutation as $\beta \to \infty$, thereby yielding UT majorization in the limit.

State transition conditions

We now have all the ingredients to derive our main result: the necessary and sufficient conditions for a state transition to be achievable through a cooling map.

Theorem 1. For two states ρ and σ on S, arbitrary except that the matrix elements of ρ are all nonzero ($\rho_{jk} \equiv \langle E_j| \rho |E_k\rangle \neq 0$), define the $d \times d$ matrix Q:

$$Q_{jk} = \begin{cases} \min\left(\frac{\sigma_{jj}}{\rho_{jj}}, 1\right), & \text{if } j = k; \\ \frac{\sigma_{jk}}{\rho_{jk}}, & \text{if } j \neq k. \end{cases} \tag{52}$$

The state transition $\rho \mapsto \sigma$ is possible through a cooling map *if and only if* both of the following conditions hold:

1. The diagonal of ρ UT-majorizes that of σ:

$$(\rho_{11} \dots \rho_{dd})^T \overset{\text{UT}}{\succ} (\sigma_{11} \dots \sigma_{dd})^T; \tag{53}$$

2. The matrix Q is positive-semidefinite:

$$Q \geq 0. \tag{54}$$

Proof$\Leftarrow$*.* Assume that the conditions stated in the theorem hold. The second condition states that $Q \geq 0$. From the first condition and Supplementary Lemma 2, it follows that the diagonal elements of Q are the diagonal elements of a UTCS matrix that maps $(\rho_{11} \dots \rho_{dd})^T \mapsto (\sigma_{11} \dots \sigma_{dd})^T$. As well, the off-diagonal elements of Q are constructed to satisfy the condition of Supplementary Lemma 1. Therefore, by the same lemma, there exists a cooling map that takes ρ to σ.

Proof$\Rightarrow$*.* Assume now that there exists a cooling map achieving $\rho \mapsto \sigma$. By Supplementary Lemma 1, there exists a $d \times d$ matrix $q \geq 0$ with the following properties:

1. The diagonal of q is also the diagonal of a UTCS matrix P such that

$$(\sigma_{11} \dots \sigma_{dd})^T = P(\rho_{11} \dots \rho_{dd})^T; \tag{55}$$

2. For $j \neq k$, $\sigma_{jk} = q_{jk}\rho_{jk}$.

Then, we have the following arguments to prove the corresponding conditions stated in the theorem:

1. From the first condition above, it follows that there exists a UTCS P that maps $(\rho_{11} \dots \rho_{dd})^T \mapsto (\sigma_{11} \dots \sigma_{dd})^T$. Therefore, by Supplementary Lemma 2,

$$(\rho_{11} \dots \rho_{dd})^T \overset{\text{UT}}{\succ} (\sigma_{11} \dots \sigma_{dd})^T. \tag{56}$$

2. Consider the matrix Q defined in the theorem statement. It has the same off-diagonal elements as q, but the diagonal elements

$$Q_{jj} = \min\left(\frac{\sigma_{jj}}{\rho_{jj}}, 1\right). \tag{57}$$

For any UTCS matrix P that maps $(\rho_{11} \dots \rho_{dd})^T \mapsto (\sigma_{11} \dots \sigma_{dd})^T$, the diagonal elements are bounded as follows:

$$P_{j|j} \leq \min\left(\frac{\sigma_{jj}}{\rho_{jj}}, 1\right). \tag{58}$$

Therefore,

$$q_{jj} = P_{j|j} \leq Q_{jj}. \tag{59}$$

This implies that

$$Q = q + D, \tag{60}$$

where D is a diagonal matrix with nonnegative entries. Since q and D are both positive-semidefinite, it follows that

$$Q \geq 0. \tag{61}$$

We can adapt the above theorem to cases where one or more ρ_{jk}'s are zero. The following proposition contains the modified version.

Supplementary Proposition 3. In cases where there are one or more zeroes in the matrix representation of ρ, the conditions of the theorem are replaced by the following revised set of conditions. In addition to the revised version of the two original conditions there is a third one, which we list *first* because it is the easiest to check (and not because we believe that any respectable theory of thermodynamics must have a "zeroth" law):

0. For each pair (j,k) such that $j \neq k$ and $\rho_{jk} = 0$, the corresponding entry in σ is also zero, i.e. $\sigma_{jk} = 0$.

1. The first of the original conditions of Theorem 1 stays the same:

$$(\rho_{11} \dots \rho_{dd})^T \overset{\text{UT}}{\succ} (\sigma_{11} \dots \sigma_{dd})^T. \tag{62}$$

2. Before we state the condition, note that the Q as defined in the theorem has diverging terms. We first take the following steps to construct an alternate *family* of Q's:

   (a) For all pairs of indices (j,k) for whom $\rho_{jk} \neq 0$, use the original definition of Q_{jk}.

   (b) For every j such that $\rho_{jj} = 0$, assign the value 0 to all Q_{jk} and Q_{kj} (i.e., to the entire j^{th} row and column).

   (c) For every pair (j,k) such that $\rho_{jk} = 0$ and Q_{jk} has not been set to zero in the previous step, allow Q_{jk} to take any value.

   The revised second condition is that *at least one* set of assignments in the last step lead to $Q \geq 0$. In this sense, instead of one specific Q, we would now have to check a range of different Q's. To minimize the complexity of this check, without loss of generality we can restrict each Q_{jk} in the last step to be real and within the interval $\left[-(Q_{jj}Q_{kk})^{1/2}, (Q_{jj}Q_{kk})^{1/2}\right]$.

Cooling maps and thermal operations

Our motivation in constructing the cooling maps model was the fact that all (low-temperature) thermal operations are cooling maps (Observation 1). Here we present some arguments that support the following conjecture:

Conjecture 1. Cooling maps are equivalent to low-temperature thermal operations, with regard to the feasibility of state transitions.

Note that this could be true even if the set of cooling maps is strictly larger than that of thermal operations—there could still be a thermal operation achieving every state transition that is possible through cooling maps.

Consider some state transition $\rho \mapsto \sigma$ that is possible under cooling maps. By Theorem 1 this corresponds to the existence of a certain $d \times d$ matrix $Q \geq 0$ associated with a possible operator sum representation of a cooling map achieving the transition. Specifically, the diagonal Kraus operators in the representation are parametrized by a collection $(\boldsymbol{\lambda}_1 \dots \boldsymbol{\lambda}_d)$ of vectors whose Gramian is Q. The ith diagonal Kraus operator contains the ith component of each of these vectors:

$$K_i = \begin{pmatrix} \lambda_1^{(i)} & 0 & \dots & 0 \\ 0 & \lambda_2^{(i)} & 0 & \vdots \\ \vdots & 0 & \ddots & 0 \\ 0 & \dots & 0 & \lambda_d^{(i)} \end{pmatrix}. \tag{63}$$

In addition, of course, there are the off-diagonal Kraus operators

$$J_{jk} = \mu_{jk} \left|j\right\rangle \left\langle k\right|, \; j < k \in \{1 \dots d\}. \tag{64}$$

If the Gramian Q has rank g, then a thermal operation implementation of $\mathcal{E}$ must necessarily use an ancilla A whose ground state has multiplicity *at least* g. Recall Eq. (14): The action of a cooling map $\mathcal{E}$ that uses an ancilla with a g-fold degenerate ground state can be written as a uniform mixture of g CPTP maps in the following manner:

$$\mathcal{E}(\rho) = \frac{1}{g} \sum_{t=1}^{g} \mathcal{E}_t(\rho), \tag{65}$$

where $\mathcal{E}_t$ is defined as

$$\mathcal{E}_t(\rho) := \mathsf{Tr}_{\mathrm{A}}\left[U\left(\rho \otimes |F_1;t\rangle\langle F_1;t|\right)U^\dagger\right]. \tag{66}$$

We can find a Kraus operator sum representation for each $\mathcal{E}_t$ using the same principle as we did before:

$$\langle E_j|\,K_{i(t)}\,|E_k\rangle := \left(\left(\langle E_j|\otimes\langle v_i|\right)U\left(|E_k\rangle\otimes|F_1;t\rangle\right)\right), \tag{67}$$

where $\{|v_i\rangle\}$ is an orthonormal basis on the space of the composite SA.

The task of finding a thermal operation implementation of $\mathcal{E}$ boils down to the task of finding a single energy-conserving U that can enable various $\mathcal{E}_t$'s, which in turn are free to be any CPTP maps as long as their uniform mixture is the channel $\mathcal{E}$. In some cases it is possible to construct a U that makes each $\mathcal{E}_t$ identical with $\mathcal{E}$, thereby realizing the latter channel overall. In such a case, the same Q is associated with all $\mathcal{E}_t$'s, but the $\boldsymbol{\lambda}$'s themselves are not required to be fixed—we only require that their Gramian be Q. The Gramian of a collection of vectors is invariant under isometries, giving us some freedom to choose the Kraus operators that we use in decomposing $\mathcal{E}$ for different t's. Let $(\boldsymbol{\lambda}_{1(t)}\dots\boldsymbol{\lambda}_{d(t)})$ be the particular vectors that we use in the tth decomposition. A U that achieves this could plausibly (although not necessarily) act in the following manner:

$$U\left(|E_k\rangle\otimes|F_1;t\rangle\right) = \left(\sum_{s=1}^{g}\lambda_{k(t)}^{(s)}\,|E_k\rangle\otimes|F_1;s\rangle\right) + \left(\sum_{j<k}\mu_{jk}\,|E_j\rangle\otimes|F_{jk};t\rangle\right), \tag{68}$$

where $F_{jk}-F_1 = E_k - E_j$, and $\{|F_{jk};1\rangle\dots|F_{jk};g\rangle\}$ may be chosen to be an orthonormal set of eigenvectors in the energy level F_{jk} (we are allowed to give arbitrary multiplicities to the energy levels of H_{A}, to suit our convenience).

The requirement that U be unitary implies that the vectors $\{U\left(|E_k\rangle\otimes|F_1;1\rangle\right)\dots U\left(|E_k\rangle\otimes|F_1;g\rangle\right)\}$ be mutually orthogonal for each k. In terms of the $\boldsymbol{\lambda}$'s, this amounts to

$$\left\langle\boldsymbol{\lambda}_{k(t)},\boldsymbol{\lambda}_{k(s)}\right\rangle \propto \delta_{ts}. \tag{69}$$

On the other hand, the Gramian of each collection $(\boldsymbol{\lambda}_{1(t)}\dots\boldsymbol{\lambda}_{d(t)})$ must be Q. This is equivalent to the requirement that these collections all be mutually connected by isometries. This condition can be phrased as a property of Q:

Property 1. For the given $d\times d$ matrix Q of rank g, there exist g sets of d vectors each, indexed as $(\boldsymbol{\lambda}_{1(t)}\dots\boldsymbol{\lambda}_{d(t)})_{t\in\{1\dots g\}}$, such that

$$\left\langle\boldsymbol{\lambda}_{j(t)},\boldsymbol{\lambda}_{k(t)}\right\rangle = Q_{jk} \tag{70}$$

for all $j,k\in\{1\dots d\}$ and $t\in\{1\dots g\}$, and

$$\left\langle\boldsymbol{\lambda}_{k(t)},\boldsymbol{\lambda}_{k(s)}\right\rangle \propto \delta_{ts} \tag{71}$$

for all $k\in\{1\dots d\}$ and $s,t\in\{1\dots g\}$.

For any Q with this property, we can construct an energy-conserving U as discussed above, therefore qualifying the associated cooling map as a (low-temperature) thermal operation.

It is easy to verify that Property 1 is possessed by any Q in the case $d=2$. Thus we have the following.

Supplementary Corollary 4. Cooling maps are equivalent to low-temperature thermal operations on two-level systems.

Recently, Ćwikliński *et al.* [5] found the conditions for two-level systems at any temperature. Our conditions match the low-temperature limit of theirs.

Another special case where Property 1 obviously follows is when Q is diagonal, and correspondingly, the final state σ in the associated thermal operation is diagonal. Therefore, the physical context of this special case is a process wherein the coherences present in the initial state are completely lost. Perhaps this is not a very useful sort of process, but the next special case lies at the opposite extreme, and is therefore—presumably—extremely useful.

If Q has rank 1, then again it is straightforward to see that Property 1 holds. In order to understand the physical significance of this special case, consider again a generic cooling map $\mathcal{E}$ with Kraus operators

$$K_i = \begin{pmatrix}\lambda_1^{(i)} & 0 & \dots & 0\\ 0 & \lambda_2^{(i)} & 0 & \vdots\\ \vdots & 0 & \ddots & 0\\ 0 & \dots & 0 & \lambda_d^{(i)}\end{pmatrix},\ i\in\{1\dots n\};$$

$$J_{jk} = \mu_{jk}\,|j\rangle\langle k|,\ j<k\in\{1\dots d\}.$$

The effect of $\mathcal{E}$ on the off-diagonal elements of states [cf. Eq. (23)] is given by

$$\rho_{jk} \mapsto \sigma_{jk} = \langle \boldsymbol{\lambda}_j, \boldsymbol{\lambda}_k \rangle \, \rho_{jk}. \tag{72}$$

By the Cauchy–Schwarz inequality,

$$\begin{aligned} \sigma_{jk} &\leq \left(\langle \boldsymbol{\lambda}_j, \boldsymbol{\lambda}_j \rangle \langle \boldsymbol{\lambda}_k, \boldsymbol{\lambda}_k \rangle \right)^{1/2} \rho_{jk} \\ &= \left(P_{j|j} P_{k|k} \right)^{1/2} \rho_{jk}, \end{aligned} \tag{73}$$

where P is the stochastic matrix governing the transformation of the diagonal elements [cf. Eq. (22)]. This bound on coherence transfer in thermal operations was also derived, for all temperatures, by Ćwikliński *et al.* in Ref. [5].

If the $\boldsymbol{\lambda}_j$'s are all pairwise linearly dependent (which is equivalent to their Gramian Q being rank-1), then the inequality is saturated for every pair (j,k). It is obvious that in such a case the "vectors" $\boldsymbol{\lambda}_j$ can be chosen to be one-dimensional (i.e., scalars) and so just one diagonal Kraus operator suffices. Therefore, of all cooling maps whose associated stochastic matrix has a given diagonal, the ones with operator sum representations comprising only one diagonal Kraus operator achieve *maximal coherence transfer* from the initial state to the final state. This motivates us to make the following definition:

Definition (Optimally coherent process). A cooling map with an operator sum decomposition consisting of exactly one diagonal Kraus operator.

The fact that Property 1 holds for such cases immediately implies

Supplementary Corollary 5. All optimally coherent processes are low-temperature thermal operations.

Ćwikliński *et al.* constructed examples of thermal processes (at general temperatures) where the bound (73) is unattainable. Our above result shows that their no-go does not hold at low temperatures, where optimal coherence transfer is always possible.

Note that every optimally coherent process achieves maximal coherence transfer *given the particular diagonal elements of the associated stochastic matrix P*. There is an additional sense in which optimization can be achieved: We can make the diagonal elements of P as large as possible. We make this idea rigorous in the following:

Supplementary Corollary 6. Let two states ρ and σ satisfy:

1. $(\rho_{11} \ldots \rho_{dd})^T \overset{\mathrm{UT}}{\succ} (\sigma_{11} \ldots \sigma_{dd})^T$;
2. The Q for the pair, as defined in Theorem 1, exists and is positive-semidefinite and rank-1.

Then,

1. There exists a thermal operation taking $\rho \mapsto \sigma$. Furthermore,
2. For any state σ' such that

$$\sigma'_{jj} = \sigma_{jj} \tag{74}$$

for all j and $\rho \mapsto \sigma'$ is possible under cooling maps, it holds that

$$\left| \sigma'_{jk} \right| \leq \left| \sigma_{jk} \right| \tag{75}$$

for every $j \neq k$.

In other words, for every pair (ρ, σ') such that $\rho \mapsto \sigma'$ is possible under *cooling maps*, $\rho \mapsto \sigma$ is possible under *thermal operations*, where σ has the same diagonal part as σ' but the *largest possible off-diagonal elements for the given diagonal* obtainable through cooling maps from the given initial state ρ.

Proof. Since ρ and σ satisfy the conditions of Theorem 1, it follows, of course, that $\rho \mapsto \sigma$ is possible through a cooling map. In fact, since the associated Q has rank 1, Property 1 holds and therefore the transition is possible through a *thermal operation*, proving the first assertion.

The rank-1 property also implies that the transition is possible by an *optimally coherent process*, therefore guaranteeing optimal coherence transfer for the given diagonal part of the associated stochastic matrix P. However, since the Q constructed in Theorem 1 has maximal diagonal elements for the given diagonal part of the final state, so does P, and the second assertion follows.

We saw that any optimally coherent process is a thermal operation, as is any "coherence-killing" process. In fact, these are both special cases of a stronger result:

Supplementary Corollary 7. Any mixture of optimally coherent processes can be approximated arbitrarily well by a thermal operation.

Proof. We will prove that any rational convex combination of optimally coherent processes is a thermal operation. By the density of the rationals among the reals, the main claim will follow.

Let a cooling map $\mathcal{E}$ be decomposable as a rational convex combination of optimally coherent processes:

$$\mathcal{E}(\cdot) = \sum_{i=1}^{n} \frac{m_i}{g} \mathcal{E}_i(\cdot), \tag{76}$$

where m_i and $g = \sum_i m_i$ are positive integers and each $\mathcal{E}_i$ is an optimally coherent process with Kraus operators

$$K_i = \begin{pmatrix} \lambda_1^{(i)} & 0 & \dots & 0 \\ 0 & \lambda_2^{(i)} & 0 & \vdots \\ \vdots & 0 & \ddots & 0 \\ 0 & \dots & 0 & \lambda_d^{(i)} \end{pmatrix};$$

$$J_{jk} = \mu_{jk}^{(i)} |j\rangle \langle k| , \ j < k \in \{1 \dots d\}.$$

To realize $\mathcal{E}$ as a thermal operation, we can use an ancilla A that has a g-fold degenerate ground energy level F_1. Let $\{|F_1; 1\rangle \dots |F_1; g\rangle\}$ be an orthonormal basis spanning this ground space. As we argued before, we can allow arbitrary degeneracies in the excited states of A and take advantage of them. We use an energy-conserving unitary U that satisfies

$$U |E_k\rangle \otimes |F_1; t\rangle = \lambda_k^{(i_t)} |E_k\rangle \otimes |F_1; t\rangle + \sum_{j<k} \mu_{jk}^{(i_t)} |E_j\rangle \otimes |F_{jk}; t\rangle , \tag{77}$$

where $i_t = 1$ for $t \leq m_1$, $i_t = 2$ for $m_1 < t \leq m_1 + m_2$, etc. Since these states are orthogonal for different t's by construction, it follows that such a unitary always exists. One may verify that the action of the resulting thermal operation on any input is identical with that of the given cooling map $\mathcal{E}$.

In the next supplementary note we will consider Gibbs-preserving operations, which in the low-temperature limit are defined by the constraint

$$\mathcal{E} \left(|E_1\rangle \langle E_1| \right) = |E_1\rangle \langle E_1| . \tag{78}$$

It is obvious that the set of low-temperature Gibbs-preserving operations is strictly larger than the set of cooling maps. Before moving on, let us summarize our findings on the various sets of operations that we have considered, through their inclusion hierarchy:

$$\begin{aligned} & \{\text{Optimally coherent processes}\} \\ \subsetneq & \{\text{Mixtures of optimally coherent processes}\} \\ \subseteq & \{\text{Low-temperature thermal operations}\} \\ \subseteq & \{\text{Cooling maps}\} \\ \subsetneq & \{\text{Low-temperature Gibbs-preserving operations}\} . \end{aligned}$$

Fig. 1 of the main text depicts a visualization of this hierarchy.

SUPPLEMENTARY NOTE 3. GIBBS-PRESERVING OPERATIONS

By constructing the cooling maps model we were able to get some elegant results about thermal operations. However, this reduction was made possible by the simplifying condition of low temperature. In general, when the temperature is arbitrary, thermal operations are not very yielding to elegant mathematical treatment, owing to their operational definition. In contrast, consider the following definition:

Definition (Gibbs-preserving operation). A quantum channel $\mathcal{E}$ that fixes the Gibbs state:

$$\mathcal{E}(\gamma_{\rm S}) = \gamma_{\rm S}. \tag{79}$$

This definition is much more mathematically direct, and so it would seem that a model wherein the allowed processes are the Gibbs-preserving operations would lend itself better to mathematical treatment. Even if one believes that such a model is not physically motivated, and rather prefers the thermal operations model, the study of the former holds some utility. From the definition of thermal operations, it is obvious that all thermal operations are Gibbs-preserving. Therefore, by studying the Gibbs-preserving model, one could potentially gain some understanding of the more challenging thermal operations model.

Here we study the low-temperature limit of the Gibbs-preserving operations, both for its own sake and in order to see how similar the results will be to the ones we obtained from cooling maps. This will give us a sense of how close the Gibbs-preserving model might be to thermal operations at higher temperatures, where we do not yet have any mathematically amenable approximation like the cooling maps.

The low-temperature approximation

Here the low-temperature limit is simpler to conceptualize than in the thermal operations case. We can define the lowness of temperature directly in terms of the system of interest S, instead of having to refer to the properties of the environment. If, as before, S is a d-level system governed by a Hamiltonian $H_{\rm S}$ with the non-degeneracy properties listed earlier, we can formalize the low-temperature assumption as follows:

$$k_B T \ll E_2 - E_1. \tag{80}$$

This leads to

$$\gamma_{\rm S} \approx |E_1\rangle\langle E_1|, \tag{81}$$

which will be the form in which we will use the approximation.

Allowed operations and the canonical parametrization

The low-temperature approximation Eq. (81) leads to the following criterion for an evolution $\mathcal{E}$ to be allowed:

$$\mathcal{E}\left(|E_1\rangle\langle E_1|\right) \approx |E_1\rangle\langle E_1|. \tag{82}$$

It is clear that the subspace spanned by $|E_1\rangle$ is treated in a privileged manner in this model. We will see this more rigorously in the upcoming sections, but in anticipation we propose the following "canonical parametrization" of a generic state of S:

$$\rho = \left(\begin{array}{c|c} \alpha & \boldsymbol{x}^\dagger \\ \hline \boldsymbol{x} & A \end{array}\right), \tag{83}$$

where $\alpha := \langle E_1|\rho|E_1\rangle \geq 0$ is a real scalar, $\boldsymbol{x}$ is a complex $(d-1)$-dimensional vector, and A is a $(d-1)$-dimensional subnormalized density operator. We can identify a state with its associated set of parameters, as $\rho \equiv (\alpha, \boldsymbol{x}, A)$.

The Schur complement construction

The following construction will be useful in the subsequent analysis. For the present, assume for simplicity that A is invertible, noting that the argument can easily be adapted to the singular case. Let

$$K_{A,\boldsymbol{x}} := \begin{pmatrix} 1 & -\boldsymbol{x}^\dagger A^{-1} \\ 0 & \mathbb{1}_{d-1} \end{pmatrix}. \tag{84}$$

The map

$$\mathcal{E}_{A,\boldsymbol{x}} : M \mapsto \mathcal{E}_{A,\boldsymbol{x}}(M) := K_{A,\boldsymbol{x}} M K_{A,\boldsymbol{x}}^\dagger \tag{85}$$

is CP. It is also invertible [1], with inverse given by the (also CP) map $\mathcal{E}_{A,-\boldsymbol{x}}$. Its action on ρ gives

$$D_\rho := \mathcal{E}_{A,\boldsymbol{x}}(\rho) = \begin{pmatrix} \alpha - \boldsymbol{x}^\dagger A^{-1}\boldsymbol{x} & 0 \\ 0 & A \end{pmatrix}. \tag{86}$$

From the CP property of $\mathcal{E}_{A,\boldsymbol{x}}$ and its inverse, it follows that $\rho \geq 0$ is equivalent to

$$\begin{aligned} A &\geq 0, \\ \alpha - \boldsymbol{x}^\dagger A^{-1}\boldsymbol{x} &\geq 0. \end{aligned} \tag{87}$$

The quantity

$$c_\rho := \alpha - \boldsymbol{x}^\dagger A^{-1}\boldsymbol{x} \tag{88}$$

is called *the Schur complement of block A in the matrix ρ.*

In order to understand how to treat cases where A is singular, note that the block A in the matrix of ρ can always be diagonalized by a unitary matrix of the form

$$U = \left(\begin{array}{c|c} 1 & 0 \\ \hline 0 & V \end{array}\right), \tag{89}$$

which is an allowed unitary under Gibbs-preserving operations. Since unitary operations are reversible, without loss of generality we can assume diagonal A in the canonical representation

$$\rho = \left(\begin{array}{c|c} \alpha & \boldsymbol{x}^\dagger \\ \hline \boldsymbol{x} & A \end{array}\right). \tag{90}$$

If a diagonal A is singular, it has some zeroes on its diagonal. But for ρ to be positive-semidefinite, the components of $\boldsymbol{x}$ in the corresponding rows must also be zero. Therefore the quantity $\boldsymbol{x}^\dagger A^{-1}\boldsymbol{x}$ can be given a well-defined value, by considering only the terms coming from the nonzero components of $\boldsymbol{x}$.

The action of allowed operations on states

Let us characterize Gibbs-preserving operations in terms of the possible Kraus operator decompositions that they can have. If an allowed channel $\mathcal{E}$ has an operator sum representation comprising the Kraus operators $\{K_1 \dots K_r\}$, the requirement of fixing $|E_1\rangle\langle E_1|$ leads to the general form

$$K_i = \left(\begin{array}{c|c} \eta_i & \boldsymbol{v}_i^\dagger \\ \hline 0 & L_i \end{array}\right). \tag{91}$$

Here $\eta_i \in \mathbb{C}$, $\boldsymbol{v}_i \in \mathbb{C}^{d-1}$, and $L_i \in \mathbb{C}^{(d-1)\times(d-1)}$. The trace-preserving condition on $\mathcal{E}$ implies that

$$\begin{aligned} \sum_i |\eta_i|^2 &= 1, \\ \sum_i \eta_i \boldsymbol{v}_i &= \boldsymbol{0}, \\ \sum_i \left(\boldsymbol{v}_i\boldsymbol{v}_i^\dagger + L_i^\dagger L_i\right) &= \mathbb{1}_{d-1}. \end{aligned} \tag{92}$$

The action of the channel $\mathcal{E}$ on a state $\rho \equiv (\alpha, \boldsymbol{x}, A)$ gives

$$\mathcal{E}(\rho) =: \sigma \equiv (\beta, \boldsymbol{y}, B), \tag{93}$$

[1] Inconveniently, the map $\mathcal{E}_{A,\boldsymbol{x}}$, while *algebraically invertible*, is *not functionally invertible*: its inversion requires information about $\boldsymbol{x}$ that is not contained in D_ρ itself!

where

$$\beta = \alpha + \sum_i \boldsymbol{v}_i^\dagger A \boldsymbol{v}_i;$$
$$\boldsymbol{y} = \left(\sum_i \eta_i^* L_i\right)\boldsymbol{x} + \sum_i L_i A \boldsymbol{v}_i;$$
$$B = \sum_i L_i A L_i^\dagger. \tag{94}$$

Recall the Schur complement construction, which associates with each state ρ a block-diagonal matrix D_ρ. Associated with the final state σ we have D_σ. The transformation from D_ρ to D_σ can be thought of as the action of the CP map

$$\Lambda_{\mathcal{E}} := \mathcal{E}_{B,\boldsymbol{y}} \circ \mathcal{E} \circ \mathcal{E}_{A,-\boldsymbol{x}}. \tag{95}$$

The action of $\Lambda_{\mathcal{E}}$ can be decomposed using the Kraus operators

$$J_i = K_{B,\boldsymbol{y}} K_i K_{A,-\boldsymbol{x}}. \tag{96}$$

We find that, by virtue of the structure of the K_i's, the J_i's have the same form:

$$J_i = \left(\begin{array}{c|c} \eta_i & \boldsymbol{u}_i^\dagger \\ \hline 0 & L_i \end{array}\right). \tag{97}$$

This leads to

$$D_\sigma = \Lambda_{\mathcal{E}}(D_\rho) = \begin{pmatrix} c_\sigma & 0 \\ 0 & B \end{pmatrix}, \tag{98}$$

where

$$c_\sigma = c_\rho + \sum_i \boldsymbol{u}_i^\dagger A \boldsymbol{u}_i. \tag{99}$$

Monotones under Gibbs-preserving operations

Monotones are real-valued functions of the state that vary monotonically (non-increasingly or non-decreasingly) under the allowed operations. For example, in classical thermodynamics, the free energy is a monotone. Here we note a couple of monotones under Gibbs-preserving operations. By virtue of the positive-semidefiniteness of the block A in the matrix of ρ, Eqs. (94) and (99) immediately yield the conditions

$$\beta \geq \alpha;$$
$$c_\sigma \geq c_\rho. \tag{100}$$

These conditions lead to the following theorem, stated in the main text with a discussion of the physical significance of the quantities involved.

Theorem 2. The quantities

$$\nu_{\mathrm{I}}(\rho) := 1 - \alpha \tag{101}$$

and

$$\nu_{\mathrm{C}} := 1 - c_\rho \tag{102}$$

are *monotonically non-increasing* under Gibbs-preserving operations.

The second monotone can be adapted to cases with singular A using the line of reasoning presented at the end of the section on the Schur complement construction.

Two-level systems and pure-state transitions

The monotones mentioned in the previous section turn out to be sufficient in determining the feasibility of state transitions in some special cases:

Supplementary Proposition 8. The conditions (100) are sufficient for state transitions on two-level systems, i.e., when $d = 2$.

Proof. In this case $A \equiv 1 - \alpha$ and $\boldsymbol{x} \equiv x$ are scalars. Similarly, among the parameters characterizing a channel $\mathcal{E}$, $\boldsymbol{v}_i \equiv v_i$ and $L_i \equiv \lambda_i$ are now scalars. For convenience we can define the following vectors:

$$\begin{aligned}\boldsymbol{\eta} &\equiv (\eta_1 \dots \eta_r)^T;\\ \boldsymbol{v} &\equiv (v_1 \dots v_r)^T;\\ \boldsymbol{\lambda} &\equiv (\lambda_1 \dots \lambda_r)^T.\end{aligned} \tag{103}$$

The TP condition Eqs. (92) can now be written elegantly:

$$\begin{aligned}\|\boldsymbol{\eta}\| &= 1;\\ \langle \boldsymbol{\eta}, \boldsymbol{v}\rangle &= 0;\\ \|\boldsymbol{v}\|^2 + \|\boldsymbol{\lambda}\|^2 &= 1,\end{aligned} \tag{104}$$

where $\langle \cdot, \cdot \rangle$ and $\|\cdot\|$ are the usual inner product and its associated geometric norm in this vector space. Under $\mathcal{E}$ [cf. Eqs. (94)], the component α transforms into

$$\begin{aligned}\beta &= \alpha + \|\boldsymbol{v}\|^2 (1-\alpha)\\ &= \alpha + \left(1 - \|\boldsymbol{\lambda}\|^2\right)(1-\alpha).\end{aligned} \tag{105}$$

The range of values that β can take under the conditions (100) is $[\alpha, 1]$, and we can always choose a $\boldsymbol{\lambda}$ that achieves any of these values while also obeying Eq. (104). It remains to be shown that any of the values of c_σ allowed by (100) can also be achieved simultaneously.

Working out the action of the channel $\Lambda_{\mathcal{E}}$ using the analysis that led to Eq. (99), we find that

$$\begin{aligned}c_\sigma &= \alpha + (1-\alpha)\|\boldsymbol{v}\|^2 - \frac{|\langle \boldsymbol{\lambda}, (x\boldsymbol{\eta} + (1-\alpha)\boldsymbol{v})\rangle|^2}{(1-\alpha)\|\boldsymbol{\lambda}\|^2}\\ &=: f[\alpha, x, \boldsymbol{v}, \boldsymbol{\lambda}, \boldsymbol{\eta}].\end{aligned} \tag{106}$$

The smallest value that c_σ can take under (100) is c_ρ. Since only the norm $\|\boldsymbol{\lambda}\|$ is relevant in achieving the requisite value of β [See Eq. (105)], we are free to choose $\boldsymbol{\lambda}$ parallel to $(x\boldsymbol{\eta} + (1-\alpha)\boldsymbol{v})$, so that

$$|\langle \boldsymbol{\lambda}, (x\boldsymbol{\eta} + (1-\alpha)\boldsymbol{v})\rangle|^2 = (\|\boldsymbol{\lambda}\| \, \|x\boldsymbol{\eta} + (1-\alpha)\boldsymbol{v}\|)^2 . \tag{107}$$

But since $\langle \boldsymbol{\eta}, \boldsymbol{v}\rangle = 0$, we have "Pythagoras' theorem":

$$\|x\boldsymbol{\eta} + (1-\alpha)\boldsymbol{v}\|^2 = |x|^2 \|\boldsymbol{\eta}\|^2 + (1-\alpha)^2 \|\boldsymbol{v}\|^2 . \tag{108}$$

This, combined with Eq. (104), gives us

$$f[\alpha, x, \boldsymbol{v}, \boldsymbol{\lambda}, \boldsymbol{\eta}] = c_\rho. \tag{109}$$

This shows that the least possible value of c_σ can be achieved. The largest possible value of c_σ is β. This can be achieved by choosing $\boldsymbol{\lambda}$ to be *orthogonal* to $(x\boldsymbol{\eta} + (1-\alpha)\boldsymbol{v})$, again without affecting the ability to achieve the desired β.

To achieve any intermediate value of c_σ, we can choose $\boldsymbol{\lambda}$ to have an intermediate direction.

Supplementary Corollary 9. The conditions (100) are sufficient when ρ and σ are both pure.

Proof. Consider a pure state

$$|\psi\rangle = t\,|E_1\rangle + \left(1 - |t|^2\right)^{1/2} |\phi\rangle\,, \tag{110}$$

where $|\phi\rangle$ is a normalized vector such that $\langle\phi|\,E_1\rangle = 0$. Using a unitary operation of the form

$$U = \left(\begin{array}{c|c} 1 & 0 \\ \hline 0 & V \end{array}\right), \tag{111}$$

which is allowed under Gibbs-preserving operations, we can always reversibly transform $|\psi\rangle$ to a state of the form

$$\left|\tilde{\psi}\right\rangle = t\,|E_1\rangle + \left(1 - |t|^2\right)^{1/2} |E_2\rangle\,. \tag{112}$$

Therefore, every state transition question involving a pair of d-dimensional pure states can be reduced to one involving pure states in the 2-dimensional subspace spanned by $\{|E_1\rangle\,, |E_2\rangle\}$. By Supplementary Proposition 8, the claim follows.

[1] Brandão, F. G., Horodecki, M., Oppenheim, J., Renes, J. M. & Spekkens, R. W. Resource Theory of Quantum States Out of Thermal Equilibrium. *Physical review letters* **111**, 250404 (2013).

[2] Horodecki, M. & Oppenheim, J. Fundamental limitations for quantum and nanoscale thermodynamics. *Nature communications* **4** (2013).

[3] Horn, R. A. & Johnson, C. R. *Matrix analysis* (Cambridge university press, 2012).

[4] Marshall, A. W., Olkin, I. & Arnold, B. C. *Inequalities: theory of majorization and its applications* (Springer, 2010).

[5] Ćwikliński, P., Studziński, M., Horodecki, M. & Oppenheim, J. Towards fully quantum second laws of thermodynamics: limitations on the evolution of quantum coherences. Preprint at `http://arxiv.org/abs/1405.5029` (2014).
